# The molecular mechanisms underlying the ERα-36-mediated signaling in breast cancer

**DOI:** 10.1038/onc.2016.415

**Published:** 2016-12-12

**Authors:** S Omarjee, J Jacquemetton, C Poulard, N Rochel, A Dejaegere, Y Chebaro, I Treilleux, E Marangoni, L Corbo, M Le Romancer

**Affiliations:** 1 Université Lyon 1, Lyon, France; 2 INSERM U1052, Centre de Recherche en Cancérologie de Lyon, Lyon, France; 3 CNRS UMR5286, Centre de Recherche en Cancérologie de Lyon, Lyon, France; 4 Department of Biochemistry and Molecular Biology, Norris Comprehensive Cancer Center, University of Southern California, Los Angeles, CA, USA; 5 Institut de Génétique et de Biologie Moléculaire et Cellulaire, Illkirch, France; 6 CNRS UMR7104, Illkirch, France; 7 INSERM U964, Illkirch, France; 8 Université de Strasbourg, Illkirch, France; 9 Centre Léon Bérard, Pathology Department, Lyon, France; 10 Institut Curie, Translational Research Department, Paris, France

## Abstract

Alterations in estrogen-mediated cellular signaling have largely been implicated in the pathogenesis of breast cancer. Here, we investigated the signaling regulation of a splice variant of the estrogen receptor, namely estrogen receptor (ERα-36), associated with a poor prognosis in breast cancers. Coupling *in vitro* and *in vivo* approaches we determined the precise sequential molecular events of a new estrogen signaling network in an ERα-negative cell line and in an original patient-derived xenograft. After estrogen treatment, ERα-36 rapidly associates with Src at the level of the plasma membrane, initiating downstream cascades, including MEK1/ERK activation and paxillin phosphorylation on S126, which in turn triggers a higher expression of cyclin D1. Of note, the direct binding of ERα-36 to ERK2 prevents its dephosphorylation by MKP3 and enhances the downstream signaling. These findings improve our understanding of the regulation of non-genomic estrogen signaling and open new avenues for personalized therapeutic approaches targeting Src or MEK in ERα-36-positive patients.

## Introduction

Estrogen signaling is essential in the initiation and development of human breast cancers. The biological actions of estrogen are mediated through estrogen receptor ERα and ERβ, which function in the nucleus in a ligand-dependent manner, composed of functional domains,^1^ including (i) the variable N-terminal A/B domain containing the transactivation domain AF-1, (ii) the C or DNA-binding domain, (iii) the hinge domain (D) and (iv) the E/F domains containing the ligand-binding domain (LBD) and the transactivation domain AF-2.^2^ Several ERα variants, derived from the alternative mRNA splicing of *ESR1* gene, have been reported,^3^ including ERα-36.^4^ The transcription of ERα-36 is initiated by a previously unidentified promoter located in the first intron of the *ESR1* gene. ERα-36 retains the DNA-binding domain, dimerization faculty and partial LBD, but lacks both AF-1 and AF-2 domains. Furthermore, the last 138 amino-acids, encoded by the final exons 7 and 8 are replaced by an extra unique 27 amino-acid sequence at the C-terminal domain (CTD). ERα-36 is mainly located at the plasma membrane and within the cytoplasm, mediating activation of the ERK pathway (for review see Rao *et al.*^5^).

The molecular mechanisms underlying estrogen signaling have been extensively studied for ERs. In addition to the well-documented effects on transcription (genomic signaling), estrogen can activate signal transduction cascades outside of the nucleus (that is, non-genomic signaling).^6,7^ In this process, ERα interacts directly with various protein kinases to form protein complexes triggering the activation of downstream molecules, such as Akt.^8,9^ One such complex is the ERα/Src/PI3K complex and our team previously demonstrated that methylation of the receptor on R260 is a prerequisite for its formation.^10^ Recently, we showed that the ERα/Src/PI3K complex is activated in aggressive breast tumors and could constitute a new potential target for therapy.^11^ In contrast, although the ERα/Src/PI3K signaling pathway is now well established, relatively little is known on the exact molecular interactions involving ERα-36. Indeed, ERα-36 was shown to activate ERK1/2 through the protein kinase C delta signaling pathway, leading to an increase in the expression of cyclin D1/cdk4, which modulates cell cycle progression.^12^ Moreover, the ERα-36 signaling pathway contributes to the potential invasion and metastasis of cancer cells,^13^ and interestingly, ERα-36 is expressed in ERα-negative breast cancer cell lines and ERα-negative tumor samples.^14, 15, 16, 17^ Intriguingly, ERα-36 can stimulate ERK activation in cells treated with the anti-estrogen tamoxifen,^17,18^ and is also involved in the development of tamoxifen resistance in ERα-positive breast cancer cell lines.^19,20^ In ERα-negative breast cancer cell lines, ERα-36 induces paclitaxel resistance through c-jun N-terminal kinases, a component of the ERK family.^21^

The aim of the present study was to unravel the entire signaling cascade conveyed by ERα-36, in order to identify potentially novel therapeutic targets or adapt existing treatments for breast cancer patients. We determined a new estrogen signaling network in ERα-negative cell lines, involving ERα-36/Src/ERK and PXN (paxillin), which, in turn, regulates cell proliferation via cyclin D1 expression. This signaling pathway was also highlighted in patient-derived xenograft (PDX) models of breast cancer treated with estrogen, confirming the importance of this signaling pathway in breast cancer.

## Results

### The functional localization of ERα-36

**Figure 1:**
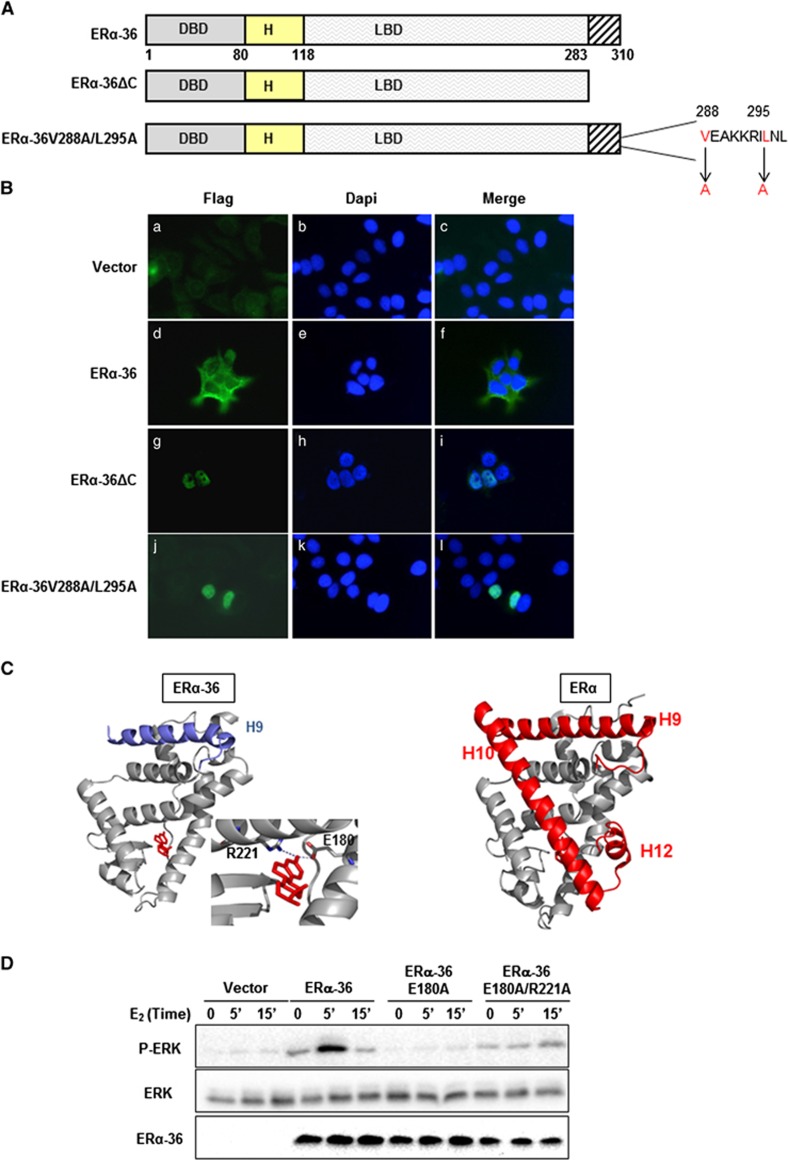
The molecular properties of ERα-36. **(A)** Schematic representation of ERα-36 mutants. ERα-36ΔC was generated by deleting the CTD. The NES mutant was obtained by mutating essential hydrophobic residues of the NES. **(B)** MCF-7 cells were transfected with pSG5Flag vector (a–c), pSG5Flag-ERα-36 (d-f), pSG5Flag-ERα-36ΔC (g-i) or pSG5FlagERα-36 V288A/L295A (j-l) for 36 h, then fixed and stained with DAPI and the anti-Flag antibody. **(C)** (Left) Modeled structure of the LBD of ERα-36 showing E_2_ docked in the ligand binding pocket. The proteins homology model is not refined and lacks the C-terminal part of ERα (H10 to H12). The specific sequence of ERα-36 is colored in blue and the ligand in red. The docked E_2_ forms electrostatic interactions with residues E180 and R221. (Right) The experimental crystal structure of ERα LBD (PDB ID 1A52,^41^) in complex with E_2_ is shown for comparison. The CTD which differs from ERα-36 is highlighted in red and the position of the helices 9, 10 and 12 are indicated. **(D)** HeLa cells were transfected with the pSG5Flag vector, the pSG5Flag-ERα-36 plasmid or the pSG5Flag-ERα-36-E180/A and pSG5Flag-ERα-36-E180/A-R221/A constructs, before E_2_ activation. A Western blot analysis detected P-ERK, ERK and ERα-36.

We initially investigated the functional localization of ERα-36, since although it contains a nuclear localization signal, it is not expressed in the nucleus, unlike ERα (see Rao *et al.*^5^). To this end, we submitted the unique CTD of ERα-36 to the NetNES 1.1 prediction server. A putative leucine-rich sequence, homologous to known nuclear export signals (NES) was identified (Supplementary Figures S1a and S1b). We then, either deleted the 27 amino-acids (ERα-36ΔC) or replaced two conserved hydrophobic residues of the NES, namely valine (V288) and leucine (L295) by alanine (A) (Figure 1A). The constructs were transfected into MCF-7 cells and analyzed by fluorescence microscopy. As expected, the wild-type (WT) ERα-36 was localized in the cytoplasm and at the level of the plasma membrane (Figure 1Bd-f), whereas the mutants were mainly detected in the nucleus (Figure 1B(g–l)). Hence, our results have uncovered the presence of a functional NES, inducing the exportation of ERα-36 from the nucleus, and able to target the nuclear arginine methyltransferase of type 1 (PRMT1) outside of the nucleus, when it is added at its N-terminus part (Supplementary Figure S1c).

### The mechanistic interaction between ERα-36 and E_2_

Since ERα-36 lacks a part of the LBD, we verified, *in silico* and *in cellulo*, whether this isoform was able to transduce estrogen-dependent signals. For this purpose, we built homology models of ERα-36 with E_2_ based on the available crystal structures of ERα LBD complexes. The initial model indicated that two residues, namely glutamic acid (E180) and arginine (R221) (corresponding to E353/R394 in ERα), could anchor the ligand in an open pocket in ERα-36 (Figure 1C). The remaining accessibility of a ligand pocket is in agreement with previous studies, confirming the high level of binding affinity between E_2_ and ERα-36.^22^ However, it must be noted that the modifications and truncation of the C-terminal helices in ERα-36 with respect to ERα may alter the conformation of the LBD, an effect that was not modelled, and further studies would be necessary to obtain a high resolution structure of ERα-36 LBD/E_2_ complex. Next, we generated point mutations of the E180 and R221 residues, replacing them with arginine. We found that overexpression of the WT protein triggered the activation of ERK upon E_2_ treatment, while the mutants lost this capacity (Figure 1D).

### ERα-36 expression in breast tumors

To further investigate the role of ERα-36 in the context of breast cancer, we produced an antibody recognizing the CTD of ERα-36. This antibody was validated by Western blot analyses using (i) GST-ERα-36, (Supplementary Figure S2a), (ii) ER isoforms transfected into HeLa cells (Supplementary Figure S2b), and (iii) CAMA-1 cells, where ERα-36 was knocked down by a siRNA approach (Supplementary Figure S2c). We then evaluated the level of expression of ERα-36 in a panel of human breast cancer cell lines, as well as in PDX models.^23^ We initially confirmed that ERα-36 is expressed both in ERα-positive and in ERα-negative breast tumors,^14, 15, 16, 17^ (Supplementary Figures S2d and S2e). One triple negative PDX, namely HBCx-12A, expressed a high level of ERα-36 (Supplementary Figures S2e and S2f), and was thus selected to establish a cell line for future experiments (named HBCc-12A) (Supplementary Figure S2g).

In HBCc-12A cells, E_2_ triggered a rapid and transient phosphorylation of ERK1/2 (Supplementary Figure S2h), though no change in Akt phosphorylation was observed. Furthermore, E_2_ led to an increase in cell proliferation *in vitro* (Supplementary Figure S2i) and *in vivo* (Supplementary Figure S2j).

### E_2_ triggers the interaction of ERα-36 with Src and PI3K kinases

**Figure 2:**
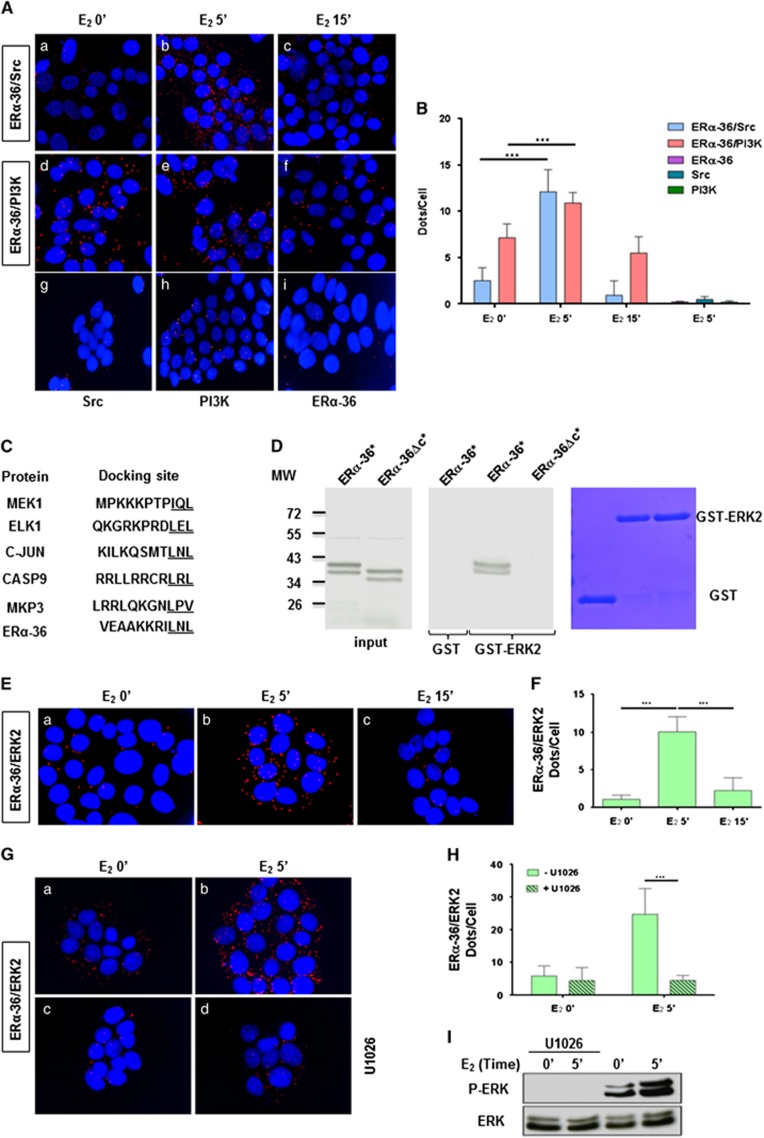
E2 triggers the interaction of ERα-36 with Src, PI3K and P-ERK2. **(A** and **B)** HBCc-12A cells were treated with E_2_ for the indicated times. (**A**) After fixation, PLA were performed to evaluate the interactions between ERα-36/Src (a–c) or between ERα-36/PI3K dimers (d–f) using ERα-36-, Src- and PI3K-specific antibodies. The detected dimers are represented by red dots. The nuclei were counterstained with DAPI (blue) (Obj: X63). Control PLA experiments were performed using single antibodies (g–i). **(B)** Quantification was performed by counting the number of signals per cell as reported in the Supplementary Material and Methods. The experiment was performed three times, and this graph is representative of one of the experiments. The *P*-value was determined using the Student's *t*-test. **(C)** Sequence alignment of known D domains in selected MAPK substrates aligned with the putative docking site. Basic residues are highlighted in boldface type, and the hydrophobic motif ΦA-X-ΦB is underlined (modified from^42^). **(D)** Direct interaction between ERα-36 and ERK2 was analyzed by GST-pull-down experiments. ^35^S-labeled *in vitro* translated ERα-36 or ERα-36ΔC was incubated with GST or GST-ERK2. 1/50 of input radiolabeled proteins were analyzed by SDS-PAGE and visualized by autoradiography. The right panel shows the corresponding Coomassie-stained gel **(E** and **F)** HBCc-12A cells were treated with E_2_. (**E**) After fixation, a PLA was performed to evaluate the ERα-36/ERK2 interaction. The nuclei were counterstained with DAPI (× 63 magnification). **(F)** The quantification of cells was performed as described in (**B**). **(G–I)** HBCc-12A cells were treated or not with the MEK inhibitor, U1026 (10 μM) for 15 min prior to E_2_ treatment. A PLA was performed to evaluate the ERα-36/ERK2 interaction. The nuclei were counterstained with DAPI (× 63 magnification). **(H)** The quantification was performed as described in (**B**). (**I**) Cell extracts were analyzed by Western blot for P-ERK and ERK expression. ***P<0.001.

Although the activation of ERK by ERα-36 was previously demonstrated,^12,17,24^ th,event were not explored. To decipher these mechanisms, we sought to identify the partners of ERα-36, by targeting known ERα partners involved in non-genomic signaling, such as Src and PI3K.^8, 10, 25^ We initially demonstrated a direct interaction between ERα-36 and both Src and PI3K (Supplementary Figures S3a and S3b), before studying protein-protein interactions using the proximity ligation assay (PLA).^26^ Upon estrogen treatment, we observed an increase in ERα-36/Src and ERα-36/PI3K interactions in the cytoplasm of HBCc-12A cells (Figure 2A(a–f)), only when using a combination of both antibodies (Figures 2A(g–i) and 2B). Since the methylation of ERα on R260 was shown to trigger its association with Src and PI3K,^10^ we verified whether such an event occurred with ERα-36, but were unsuccessful (data not shown). Furthermore, since Src and PI3K kinase activities are required for their interaction with ERα,^11^ we investigated whether they were also required to interact with ERα-36. Treatment of HBCc-12A cells with the Src inhibitor PP1 only abolished the ERα-36/Src interaction, while the PI3K inhibitor LY294002 only inhibited the ERα-36/PI3K interaction (Supplementary Figures S3c and S3d).

In conclusion, ERα-36 interacts with Src and PI3K but the mechanism involved seems to be different from ERα.

### ERα-36 binds specifically to P-ERK2

**Figure 3:**
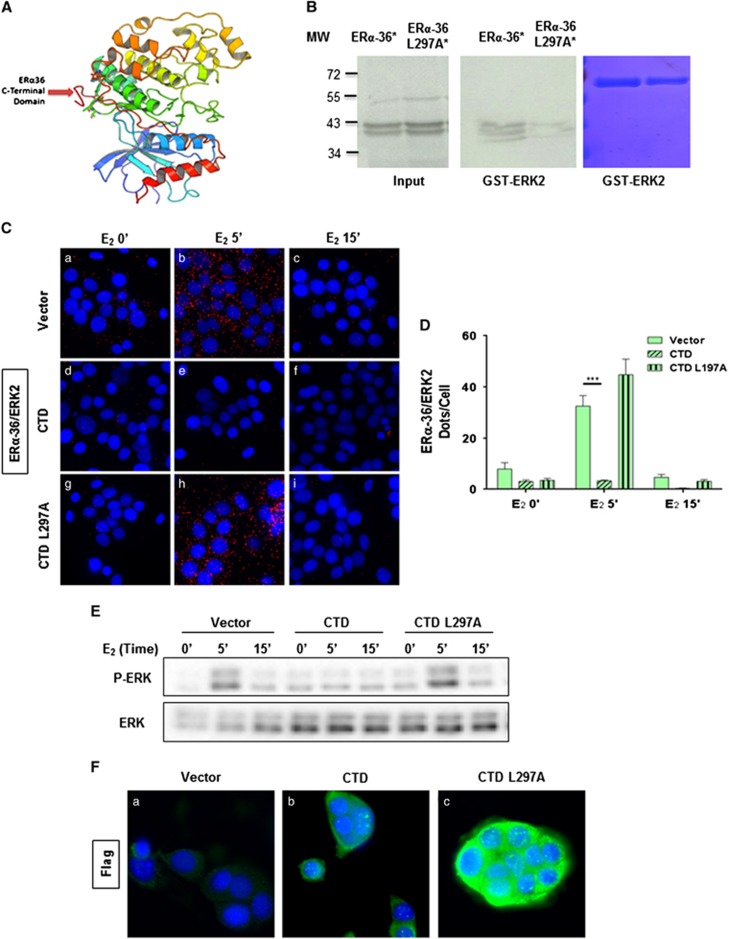
Disruption of ERα-36/ERK2 interaction abolishes E_2_-induced ERK activation. **(A)** Modeled structure of the ERK2 complex with the CTD of ERα-36. The structure of ERK2 is represented as ribbons, and based the PDB ID 2FYS. The modeled CTD of ERα-36 is represented in red. **(B)** To study the importance of L297 on ERα-36/ERK2 interaction, a GST pull-down experiment was performed by incubating the GST-ERK2 in the presence of *in vitro* translated ^35^S-labeled ERα-36 or ERα-36-L297A mutant (*). 1/50 of input radiolabeled proteins were analyzed by SDS-PAGE and visualized by autoradiography. The corresponding Coomassie-stained gel is shown in the right panel. **(C–F)** The role of the CTD of ERα-36 was investigated by transfecting the HBCc-12A cells were transfected with pSG5-Flag vector, pSG5-Flag-ERα-36-CTD or pSG5-Flag- ERα-36-CTD-L297A for 36 h. The cells were treated with E_2_ and fixed in methanol. (**C**) ERα-36/ERK2 interactions were analyzed by PLA. The nuclei were counterstained with DAPI (blue) (× 63 magnification). **(D)** Quantification of the cells was performed as described in Figure 2B. The experiment was performed in triplicate, and this graph is representative of one of the experiments. The *P*-value was determined using the Student's test. ***P<0.001. (**E**) P-ERK and ERK were analyzed by Western blot. **(F)** To verify the efficay of transfection, the cells were fixed and stained with DAPI and anti-Flag antibody.

To identify ERα-36-specific partners, the use of the Scansite software for the CTD uncovered a putative D domain (docking domain) for ERK2 (Figure 2C). We verified the interaction of ERα-36 and ERK2 by GST pull-down and found that this occurs via the CTD (Figure 2D and Supplementary Figure S4a), and *in cellulo* experiments revealed that this interaction occurs very rapidly in the cytoplasm of the HBCc-12A cells upon the addition of E_2_ (Figures 2E and F; Supplementary Figure S4b). The use of the MEK inhibitor U1026 abolished the ERα-36/ERK2 interaction (Figures 2G and H), concomitantly with ERK phosphorylation (Figure 2I), suggesting that ERα-36 interacts specifically with P-ERK2. Finally, *in vitro* phosphorylation assays revealed that ERα-36 is not a substrate for ERK2 (data not shown). An analysis of the 3D structure of ERK2-peptide complexes (Supplementary Table S3 for the list of the complexes) consistently highlighted the essential role of R and L residues in stabilizing the complexes (Supplementary Table S1). Comparison of these data with the ERα-36 CTD sequence demonstrated the implication of a leucine (L297) residue in this interaction, and indicated that the CTD could adopt a structure similar to that seen in existing complexes (Figures 3A for a 3D *in silico* model representing a putative interaction between the CTD of ERα-36 and ERK2 and Supplementary Table S1). The point mutation of this residue with alanine resulted in an impairment in the interaction (Figure 3B). Furthermore, we disrupted the ERα-36/ERK interaction by transfecting HBCc-12A cells with a Flag-CTD of WT ERα36 or mutated on L297A. We found that the Flag-CTD strongly impaired the E_2_-induced ERα-36/ERK2 interaction (Figures 3C(d–f) and 3D) compared to the empty vector (Figure 3C(a–c)). Interestingly, the Flag-CTD peptide also completely disrupted ERK phosphorylation (Figure 3E). These effects were not observed in cells transfected with the CTD mutant (Figures 3C(g–i) and 3E). Control immunofluorescence (IF) experiments revealed that Flag-CTD peptides were equally expressed (Figure 3F).

Taken together these data revealed that the CTD of ERα-36 is essential for ERK phosphorylation.

### ERα-36 protects from ERK dephosphorylation by the phosphatase MKP3

**Figure 4:**
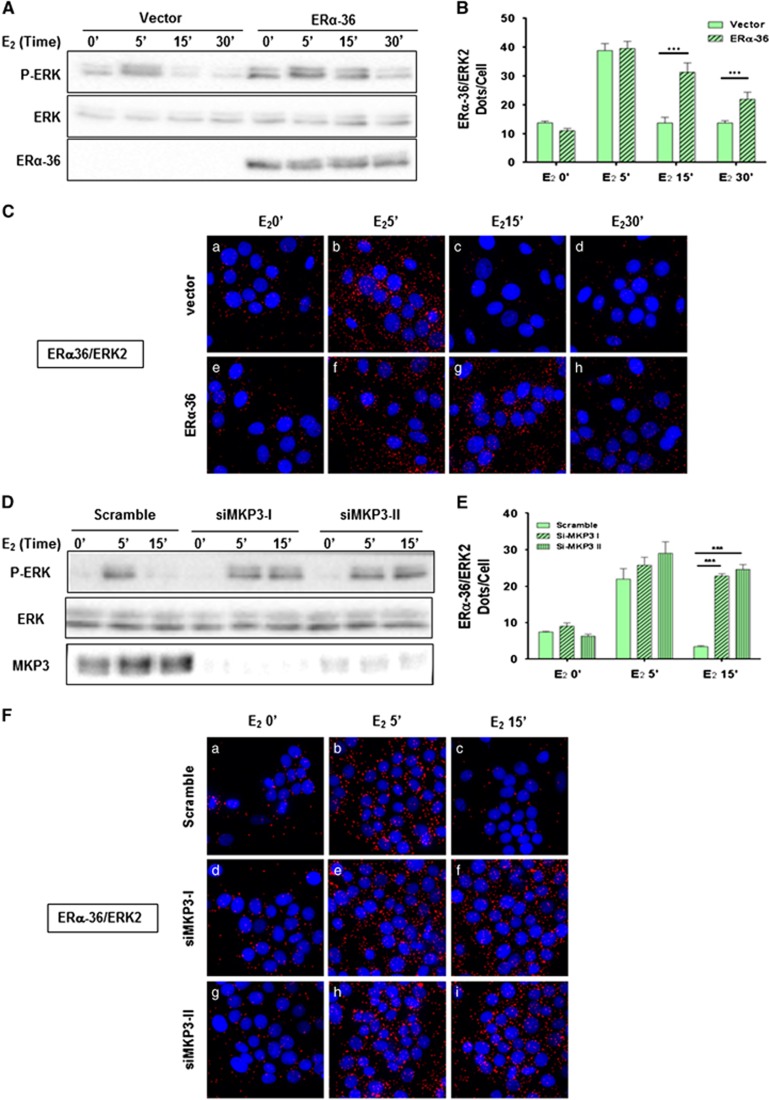
Crosstalk between ERα-36 and MKP3 to regulate ERK phosphorylation. **(A–C)** To determine the role of ERα-36 on ERK signaling, the pSG5-Flag or pSG5-Flag-ERα-36 vectors were transfected into HBCc-12A cells for 36 h prior to E_2_ activation. (**A**) The cell extracts were analyzed for the expression of P-ERK, ERK and ERα-36. **(B)** ERα-36/ERK2 interactions were quantified as described in Figure 2C. The experiment was performed in triplicate, and this graph is representative of one of the experiments. The *P*-value was determined using the Student's test. ***P<0.001. **(C)** Cells were used to perform PLA to detect, ERα-36/ERK2 interactions. **(D–F)** To investigate if MKP3 was the phosphatase involved in regulating ERK phosphorylation, we transfected MCF-7 cells with control siRNA duplexes or with specific MKP3 siRNA duplexes. Next, cell lysates were analyzed for P-ERK, ERK and MKP3 expression. **(E)** Quantification of the interactions was done as described in (**B**). (**F**) Cells were used to perform PLA, as described in (**C**), to detect ERα-36/ERK2 interactions.

**Figure 5:**
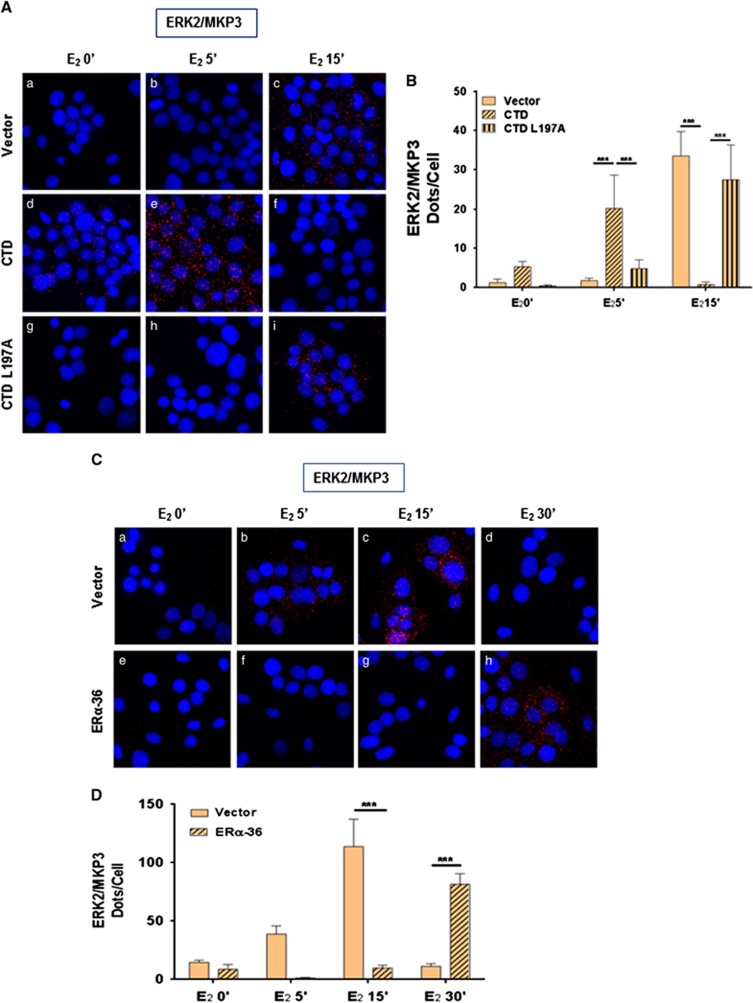
ERα-36 impedes the interaction between MKP3 and ERK2. To study the role of ERα-36 on ERK2/MKP3 interaction, we overexpressed the CTD of ERα-36 (**A** and **B**) or ERα-36 (**C** and **D**). **(A** and **B)** HBCc-12A cells were transfected with pSG5-Flag vector, pSG5-Flag-ERα-36-CTD or pSG5-Flag-ERα-36-CTD-L297A prior to E_2_ treatment. (**A**) A PLA was then conducted to analyze ERK2/MKP3 interactions (× 63 magnification). **(B)** Quantification was performed as described in Figure 2B. The experiment was performed in triplicate, and this graph is representative of one of the experiments. The *P*-value was determined using the Student's test. ***P<0.001. **(C** and **D)** The pSG5-Flag and pSG5-Flag-ERα-36 vectors were transfected into HBCc-12A cells for 36 h prior E_2_ treatment. (**C**) We then analyzed ERK2/MKP3 interactions by PLA as described in (**A**). (**D**) Quantification was performed as described in Figure 2B.

We then overexpressed ERα-36 in HBCc-12A cells and found a sustained ERK phosphorylation (beyond 15 min) upon E_2_ activation (Figure 4A), concomitantly with a sustained ERα-36/ERK2 interaction (Figures 4B and C(e–h)), compared to cells transfected with an empty vector (Figure 4C(a–d)). This effect is specific for ERα-36, since the overexpression of ERα does not have any effect on ERK phosphorylation (Supplementary Figure S5). We hypothesized that ERα-36 could regulate ERK phosphorylation by modulating its dephosphorylation. Since the dual phosphatase MKP3 regulates ERK phosphorylation,^27^ we knocked down MKP3, and found a sustained ERK phosphorylation upon E_2_ treatment, demonstrating that MKP3 depletion was sufficient to inhibit ERK dephosphorylation (Figure 4D). This event occurred concomitantly with a sustained ERα-36/ERK2 interaction (Figures 4E and F). We then hypothesized that the binding of ERK2 to ERα-36 could impede its binding to MKP3. In order to verify this hypothesis, we studied ERK2/MKP3 interaction by PLA in HBCc-12A cells transfected with the different Flag-CTD constructs. Interestingly, we found that upon E_2_ treatment, ERα-36 binds to ERK2 after 5 min while MKP3 binds to ERK2 after 15 min. However, when we disrupted the ERα-36/ERK2 interaction, MKP3 bound to ERK2 within 5 min of treatment (Figures 5A and B). The Flag-CTD L297A mutant displayed similar results to those obtained with the empty vector. Finally, when we overexpressed ERα-36 in HBCc-12A cells (as in Figure 4A), we observed that the ERK2/MKP3 interaction occurred later than in the control cells (Figures 5C and D). Overall, these findings confirm our initial hypothesis that ERα-36 prevents and delays the ERK2/MKP3 interaction, thus leading to a sustained phosphorylation of ERK2.

### The E_2_/ERα-36 pathway, downstream and upstream of P-ERK

**Figure 6:**
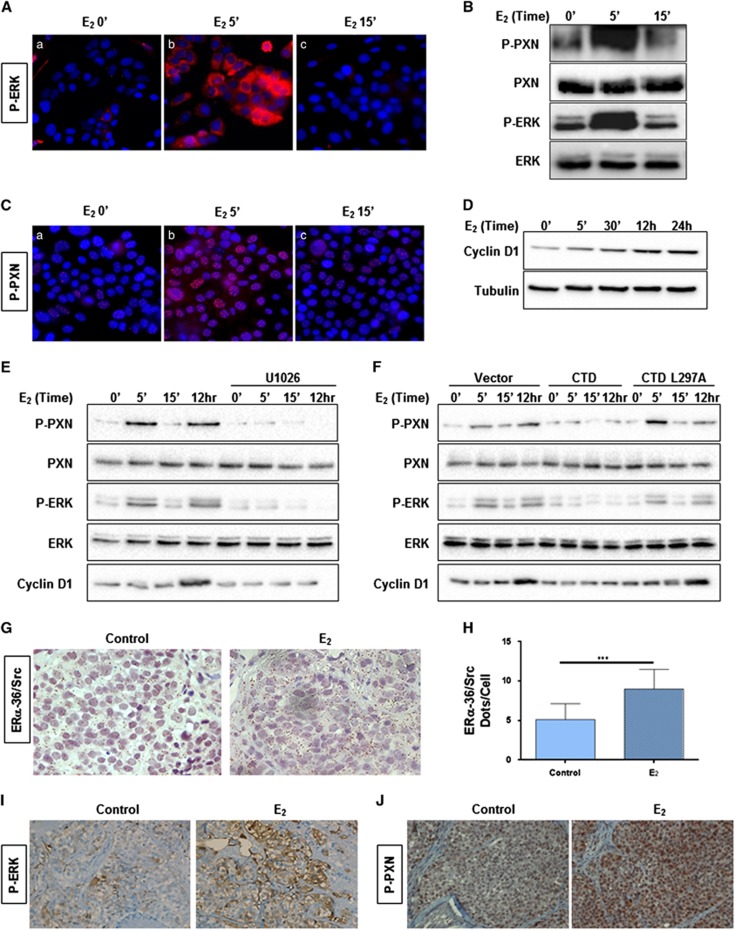
The estrogen signaling pathway downstream of ERK. **(A)** P-ERK localization was assesed upon E2 treatment. HBCc-12A cells were treated with E_2_, then fixed and immunostained with the anti-PERK antibody by IF (× 63 magnification). **(B)** From the same treatment, cells were lyzed and the cell extracts were subsequently analyzed by Western blot for the expression of P-ERK, ERK, P-PXN and PXN. **(C)** The cells treated in A were fixed and immunostained to study the localization of P-PXN. **(D)** HBCc-12A cells were treated with E_2_ for longer time to study cyclin D1 expression. The extracts were analyzed by Western blot for the expression of cyclin D1 and tubulin. **(E)** To study the role of ERK phosphorylation on cyclin D1 expression, HBCc-12A cells were treated or not with the MEK inhibitor, U1026 (10 μM) for 15 min prior to E_2_ treatment. Expression of P-ERK, ERK, P-PXN, PXN, cyclin D1 and tubulin were assessed by Western blot. **(F)** Next, we investigated the role of ERα-36 on ERK signaling. To this aim, HBCc-12A cells were transfected with pSG5-Flag vector, pSG5-Flag-ERα-36-CTD or pSG5-Flag- ERα-36-CTD-L297A for 36 h. The cell extracts were analyzed by Western blot for P-ERK, ERK, P-PXN, PXN and cyclin D1 expression **(G–J)** HBCx-12A PDX from which HBCc-12A cells were derived was grown with and without the supplementation of E_2_ in the drinking water of mice (see Supplementary Appendix Figure S2i). Mice were killed at the end of the experiment (day 51) and tumours were embedded in paraffin. (**G**) A bright field PLA was performed to study ERα-36/Src interactions in each group. The brown dots represent protein-protein interactions (× 40 magnification). **(H)** The interactions were quantified as described in Figure 2B. The *P*-value was determined using the Student's test. ***P<0.001. **(I)** On the same embedded tumors, P-ERK was also assessed by IHC staining. **(J)** P-PXN was assessed by IHC staining.

Upon E_2_ treatment, P-ERK remains exclusively in the cytoplasm of the cells (Figure 6A), so the adaptor paxillin (PXN), which is a cytoplasmic substrate for ERK, was a good candidate.^28^ We found that upon E_2_ treatment, the phosphorylation on residue S126 increased rapidly following ERK activation (Figure 6B). We then verified its subcellular localization upon E_2_ treatment, and found that P-S126-PXN was present exclusively within the nucleus (Figure 6C). It was previously reported that P-S126-PXN can participate in the transcriptional regulation of cyclin D1.^29^ We therefore studied the expression of cyclin D1 following E_2_ treatment and observed an increase in cyclin D1 expression after 12 h (Figure 6D). Interestingly, when the pathway was blocked using the MEK inhibitor or when the ERα-36/ERK2 interaction was disrupted, E_2_ failed to induce PXN phosphorylation and cyclin D1 expression (Figures 6E and F).

Next, we investigated whether the pathway occurred in the original PDX from which the HBCc-12A were derived (HBCx-12A) grown in the presence of E_2_. We found an increase in ERα-36/Src interaction (Figures 6G and H), P-ERK (Figure 6I) and P-PXN (Figure 6J). Unfortunately, we were unable to detect ERα-36/ERK2 interaction possibly due to the fact that ERK2 antibodies may not work in PLA conducted on formalin-fixed tissues. In addition, this pathway was investigated in the ERα-negative HBL100 cell line, and we observed that E_2_ triggered ERα-36/ERK2 interaction, ERK activation, PXN phosphorylation, as well as cell proliferation (Supplementary Figures S6a–d).

**Figure 7:**
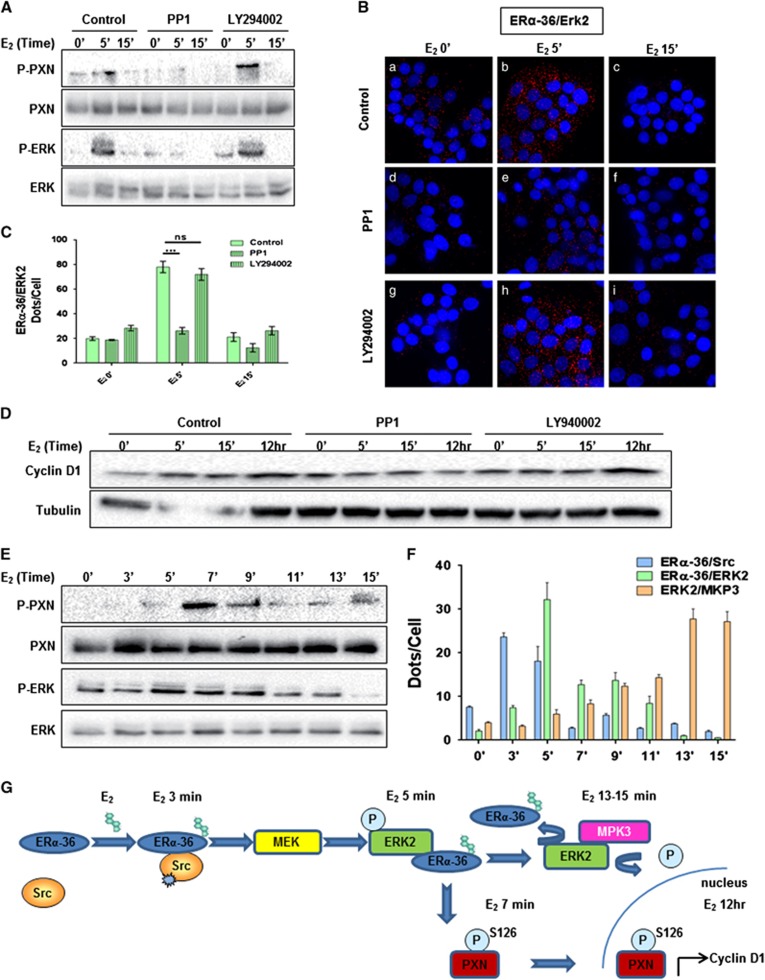
Src activity regulates E_2_-induced ERK signaling. **(A–D)** To study the role of Src and PI3K on E2-induced ERK signaling, HBCc-12A cells were treated or not with PP1 (5 μM) or LY294002 (20 μM) for 15 min before E_2_ treatment. (**A**) Cell lysates were analyzed by Western blot for the expression of P-ERK, ERK, P-PXN and PXN. **(B)** Cells were also used to perform PLA to detect ERα-36/ERK2 interactions (× 63 magnification). **(C)** Quantification of the interactions was performed as in Figure 2B. The experiment was performed in triplicate, and this graph is representative of one of the experiments. *P*-value was determined by the Student's test. ***P<0.001. **(D)** HBCc-12A cells were treated as described in (**A**) but for a longer period to study cyclin D1 expression. **(E** and **F)** HBCc-12A cells were treated with E_2_, cell lysates were analyzed by Western blot for the expression of P-ERK, ERK, P-PXN and PXN. **(F)** ERα-36/Src, ERα-36/ERK2 and ERK2/MKP3 interactions were studied by PLA (Supplementary Figure S7), the quantifications were performed as described in Figures 2B. (**G**) E_2_/ERα-36 signaling pathway.

We then wondered whether Src and PI3K activities occured upstream of the ERK pathway, and found that only PP1 treatment completely abolished E_2_-induced ERK and PXN phosphorylation (Figure 7A), ERα-36/ERK2 interaction (Figures 7B and C, compare panels d–f to control experiments panels a–c), as well as cyclin D1 expression (Figure 7D). LY294002 had no effect on these activities (Figures 7A and B(g–i) 7C and 7D). Finally, the kinetic of the various events of this signaling pathway was determined by treating HBCc-12A cells with E_2_ for a very short period of time. We unveiled that (i) ERα-36 associates with Src within 3 min of E_2_ treatment then dissociates after 5 min (Figure 7F and Supplementary Figure S7), (ii) ERα-36 binds to ERK2 with a peak occurring after 5 min of treatment, concomitantly with ERK and PXN phosphorylation (Figure 7E), and (iii) after 11 min, MKP3 starts binding to ERK2 and the interaction until 15 min (Figure 7F and Supplementary Figure S7).

## Discussion

Approximatively 80% of breast cancers express ERα and endocrine therapies have led to significant improvements in patient survival. In contrast, triple-negative breast cancers, which are negative for ERα, for the progesterone receptor and for the human epidermal growth factor receptor 2, are highly aggressive and treatment options are, so far, restricted to cytotoxic agents.^30^ In this study, focusing on triple negative breast cancers, we unveiled the precise molecular events underlying ERα-36-mediated signaling pathway. These findings introduce a new paradigm in which ERα-36 activates ERK signaling at two levels, at the plasma membrane by binding to Src and in the cytoplasm by directly binding to P-ERK.

Similarly to ERα, we observed the binding of ERα-36 with Src and PI3K; however, the mechanisms underlying the formation of the complex seem different. Indeed, the ERα/Src interaction was dependent on the methylation of ERα on residue R260 (corresponding to R184 in ERα-36),^10^ as well as on its phosphorylation on the Y537 residue, a docking site for the SH2 domain of Src.^31^ Here, we were unable to detect any ERα-36 methylation on R184, and, in addition Y537 was not present in ERα-36. We can speculate that a yet unidentified tyrosine residue could be involved in their interaction.

The functional activation of ERK by ERα-36 was shown by various groups.^12,17,24^ In the present study, we mechanistically demonstrated that two routes tightly regulate ERK2 activation by acting both on its phosphorylation and dephosphorylation. We described for the first time a direct binding of ERα-36 with ERK2 via a newly identified D domain located in its CTD. The D domain is a conserved docking motif in MAPKs used in the recognition of their activators, substrates and regulators, such as kinases and phosphatases.^32^ A combined *in silico* 3D model and *in vitro* approach enabled us to identify the crucial role of L297 in the binding interaction between ERα-36 and ERK2, which was confirmed since its point mutation completely disrupted the interaction with ERK2 (Figure 3B). Deactivation of ERK1/2 is carried out by several serine threonine or tyrosine phosphatases, including MKP3, a dual phosphatase with the capacity to dephosphorylate both threonine and tyrosine residues.^33,34^ Interestingly, we found that ERα-36 was capable of preventing binding of MKP3 to ERK presumably through stearic competition, maintaining the activation of ERK. This was confirmed by disrupting the ERα-36/ERK2 interaction, allowing a more rapid binding of MPK3, and resulting in the constant dephosphorylation of ERK. Such a regulation of P-ERK has already been described for the adaptor molecule MyD88 and the Rab2A GTPase. Indeed, MyD88 and Rab2A GTPase prevent ERK inactivation by MKP3, leading to cell transformation^35^ and to promoting breast cancer stem cells, respectively.^36^

Although many substrates for ERK are localized in the nucleus, others are found in the cytosol.^37^ PXN is a 68-kDa focal adhesion-associated protein, which functions as a scaffolding protein assembling signaling molecules into complex downstream of integrin and growth factors, regulating matrix organization, cell motility and proliferation.^38,39^ Initially, ERK was identified as a priming kinase for the GSK3-mediated PXN phosphorylation on residue S126.^28^ Furthermore, PXN was shown to function as an upstream mediator of ERK activation and a downstream regulator of ERK signaling via different phosphorylations. The epidermal growth factor and dihydrotestosterone (a ligand to Androgen receptor) induce Src-mediated phosphorylation of PXN on residue Y118 to activate ERK phosphorylation but also trigger ERK-mediated phosphorylation of PXN on residue S126.^40^ Since we demonstrated that P-PXN translocates to the nucleus following E_2_ treatment (Figure 6C), and that ERα-36 triggers cyclin D1 transcription,^20^ we speculated that PXN activates cyclin D1, thus triggering cell proliferation (Figure 6D).

Based on our results, we propose the following model of regulation of the E_2_/ERα-36 signaling pathway (Figure 7G). When E_2_ enters within the cells, it causes the interaction of ERα-36 with Src, inducing MEK activation, which in turn phosphorylates ERK. Activated ERK phosphorylates its substrate PXN on S126, triggering its translocation to the nucleus where it acts as a coactivator to induce cyclin D1 transcription, and increasing cell proliferation. Interestingly, ERα-36 reinforces the signal induced by the P-ERK phosphorylation by preventing its rapid dephosphorylation by MKP3.

The identification of this new signaling pathway could have significant implications in breast cancer treatment. Indeed, a retrospective study of 896 cases of breast cancer patients revealed that 40% cases of ERα-positive breast cancers expressed ERα-36, and those patients were less inclined to benefit from tamoxifen therapy.^16^ Moreover, 40% of ERα-negative breast cancers, while lacking ERα expression, expressed ERα-36. Its prognostic value in this breast cancer subtype is less clear (for a review see Gu *et al.*^14^). Future studies could determine whether this novel pathway is conserved in ERα-positive and -negative subtypes and for ERα-36-positive tumors, while combining Src or MEK inhibitors with hormonotherapy may improve the response to conventional treatments.

## Materials and methods

### Antibodies

A polyclonal antibody against ERα-36 specifically generated for this study by Covalab (Lyon, France), and commercially-available antibodies are listed in Supplementary Table S2.

### Cell culture

MCF-7 and HeLa cells were obtained from ATCC. The HBCc-12A cell line was established from the HBCx-12A xenograft, a PDX model of primary triple-negative breast cancer.^23^ Additional information can be found in the Supplementary Materials and Methods.

### Plasmids and mutagenesis

The pCDNA3-ERα-36 plasmid was a gift from Dr Wang.^4^ The mutations were obtained using the Quickchange XL Site-Directed Mutagenesis Kit according to the manufacturer's instructions (Agilent Technologies, Santa Clara, CA, USA). Additional information can be found in the Supplementary Materials and Methods.

### Proximity ligation assay (PLA)

This technology developed by Olink Bioscience (Sweden) enables the visualization of protein/protein interactions *in situ* and was firstly published in 2006.^26^ Additional information can be found in the Supplementary Materials and Methods.

### Molecular modeling

Homology models of ERα-36 LBD (118-310) were obtained using the structure prediction server ROBETTA (http://robetta.bakerlab.org/). The amino-acid sequence of ERα-36 was retrieved from NCBI (GenBank: BX640939.1). 3D Models were compared to the crystal structures of ERα-E_2_ (PDB ID: 1A52). Analysis of the complexes between ERK2 and D motif peptides was performed as described in the Supplementary Materials and Methods. Figures were realized with the PyMOL molecular graphics software.
