# Supplementary material for: The molecular mechanisms underlying the ERα-36-mediated signaling in breast cancer
Source: Oncogene. 2016 Dec 12;36(18):2503–14. doi: 10.1038/onc.2016.415 (PMC5422711; doi:10.1038/onc.2016.415)
Supplement: Supplementary Data [file onc2016415x1.pdf]

Supplementary Information

The molecular mechanisms underlying the ERα-36-mediated signaling in breast cancer

Soleilmane Omarjee[1,2,3], Julien Jacquemetton,[1,2,3], Coralie Poulard[1,2,3,4], Natacha Rochel[5,6,7,8], Annick Dejaegere [5,6,7,8] , Yassmine Chebaro[5,6,7,8], Isabelle Treilleux [1,2,3,9] , Elisabetta Marangoni[10], Laura Corbo[1,2,3] and Muriel Le Romancer[1,2,3]

Supplemental Figures

a

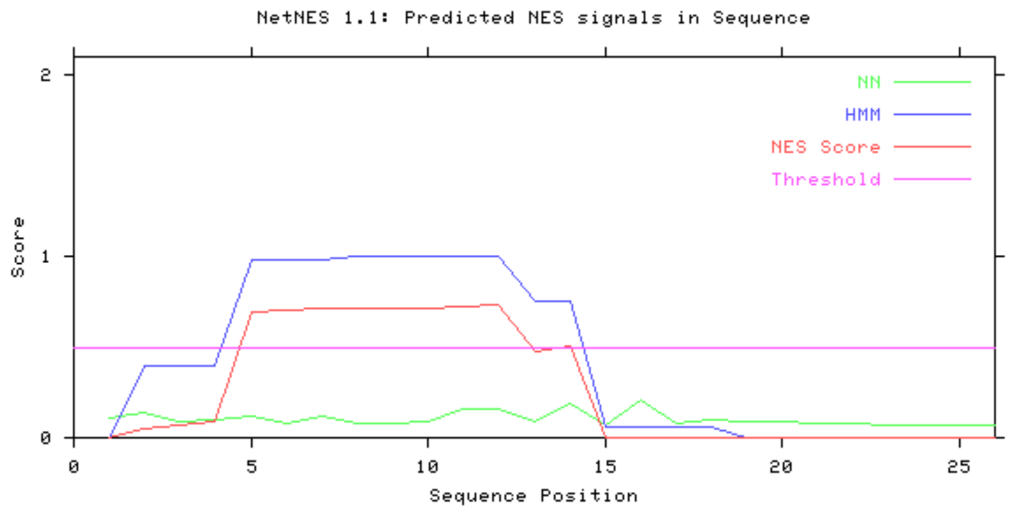

1 GISHVEAKKRILNLHPKIFGNKWFPRV 27

b

Protein	NES sequence
PKI	LALKLAGLDI
MAPKK	LQKKLEELEL
NMD3	LAEMLEDLHI
TFIIIA	L-PVLENLTL
ERα-36	VEAKKRILNL
NES consensus	$\phi X_{2-3}\phi X_{2-3}\phi X\phi$

c

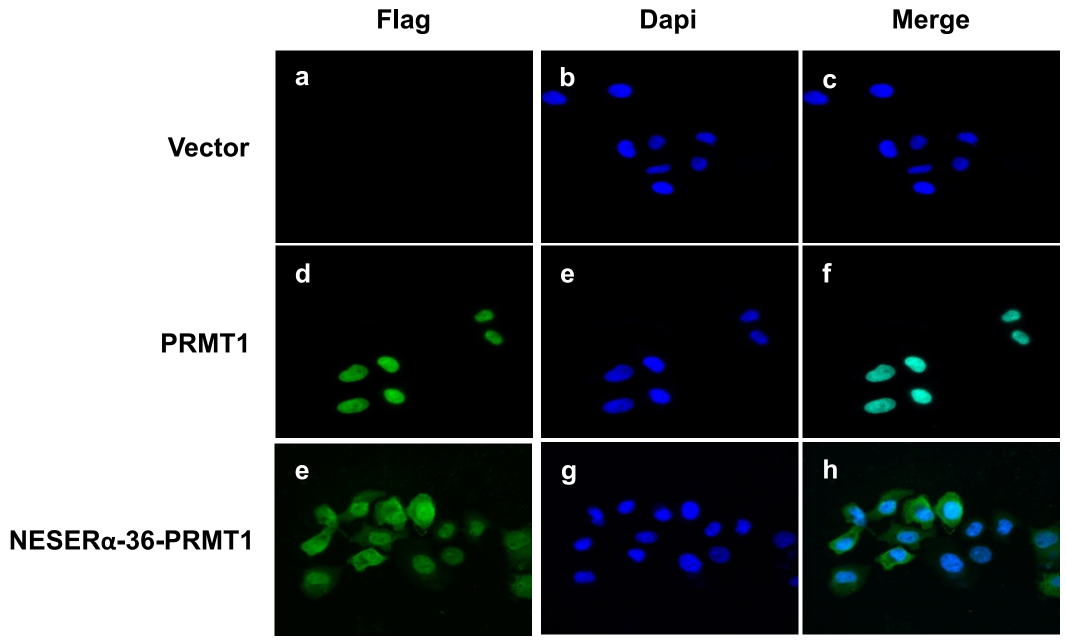

Figure S1

Supplementary Figure 1. Identification of a nuclear export signal in ERα-36 sequence

(a) The NetNES algorithm predicts a putative leucine-rich NES encoded by exon 9 in ERα-36. Scores obtained from the neural network (NN) and hidden Markov model (HMM) calculations are plotted in green and blue, respectively. The combined score obtained from the NetNES algorithm is plotted in orange, and the cut-off threshold is shown as a pink line. Only the portion of the sequence with scores above this threshold is shown.
(b) The putative ERα-36 NES sequence shows a high level of conservation with other known CRM1-dependent NES, following their alignment (modified from Goulet et al., 2007). The conserved hydrophobic residues are shown in red.
(c) MCF-7 cells were transfected with the pSG5Flag vector (panels a–c), pSG5Flag-PRMT1 (panels d-f) or pSG5-NESERα-36-Flag-PRMT1 (panels g-i), for 36 hr, then fixed and stained with DAPI and the anti-Flag antibody.

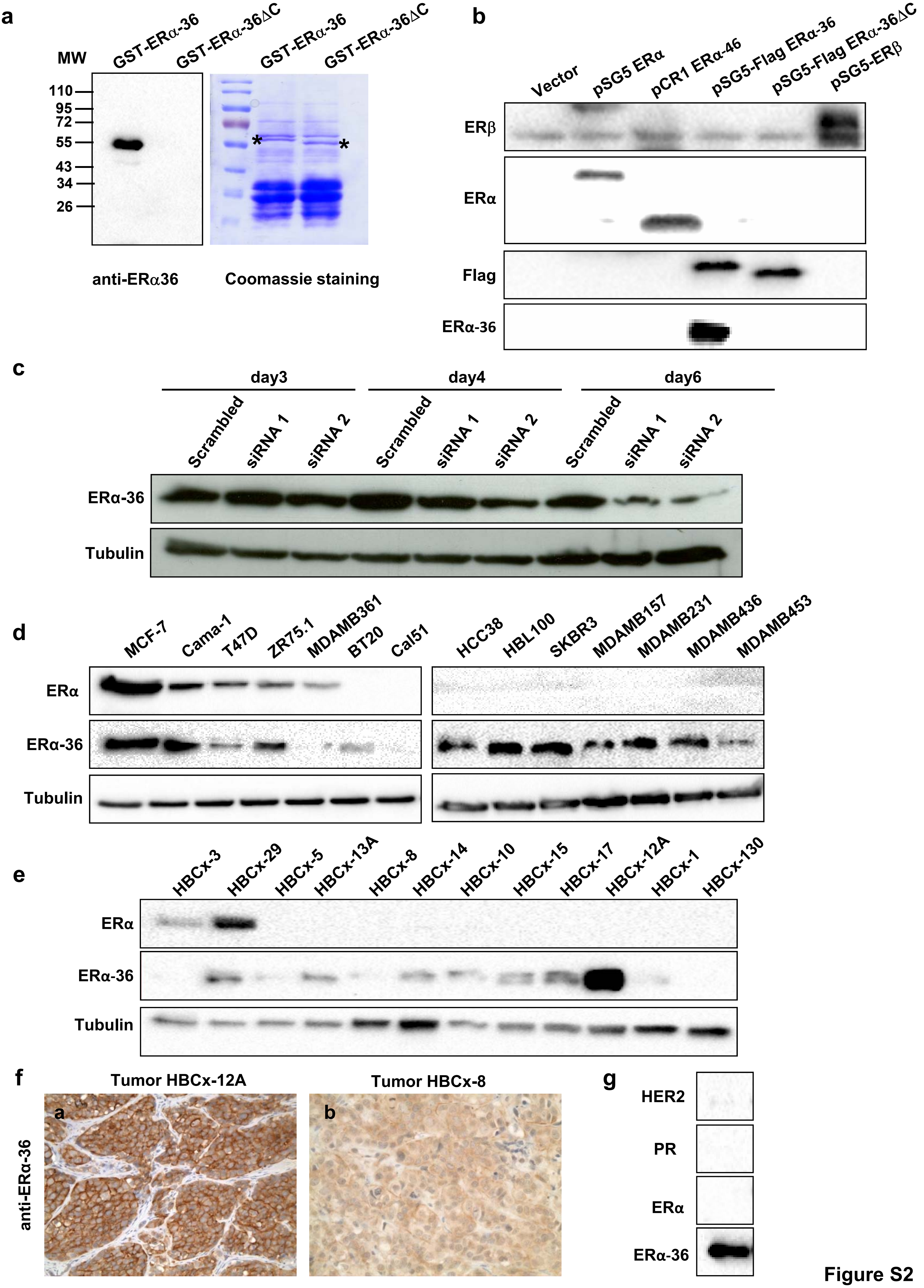
a
MW
GST-ERα-36
GST-ERα-36ΔC
GST-ERα-36
GST-ERα-36ΔC
110
95
72
55
43
34
26
*
*
anti-ERα36
Coomassie staining
b
Vector
pSG5 ERα
pCR1 ERα-46
pSG5-Flag ERα-36
pSG5-Flag ERα-36ΔC
pSG5-ERβ
ERβ
ERα
Flag
ERα-36
c
day3
day4
day6
Scrambled
siRNA 1
siRNA 2
Scrambled
siRNA 1
siRNA 2
Scrambled
siRNA 1
siRNA 2
ERα-36
Tubulin
d
MCF-7
Cama-1
T47D
ZR75.1
MDAMB361
BT20
Cal51
HCC38
HBL100
SKBR3
MDAMB157
MDAMB231
MDAMB436
MDAMB453
ERα
ERα-36
Tubulin
e
HBCx-3
HBCx-29
HBCx-5
HBCx-13A
HBCx-8
HBCx-14
HBCx-10
HBCx-15
HBCx-17
HBCx-12A
HBCx-1
HBCx-130
ERα
ERα-36
Tubulin
f
Tumor HBCx-12A
Tumor HBCx-8
a
b
anti-ERα-36
g
HER2
PR
ERα
ERα-36

Figure S2

h

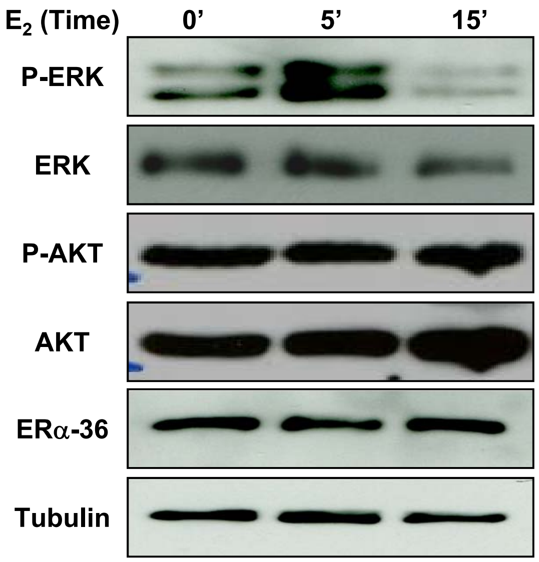

i

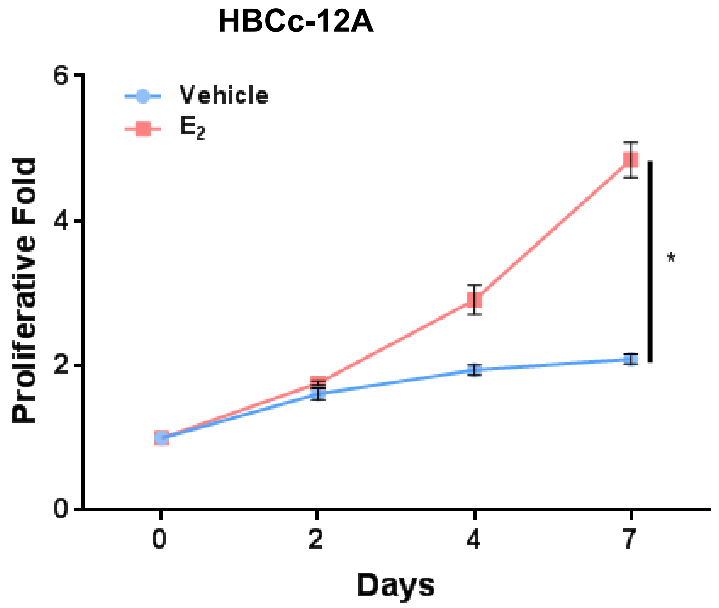

j

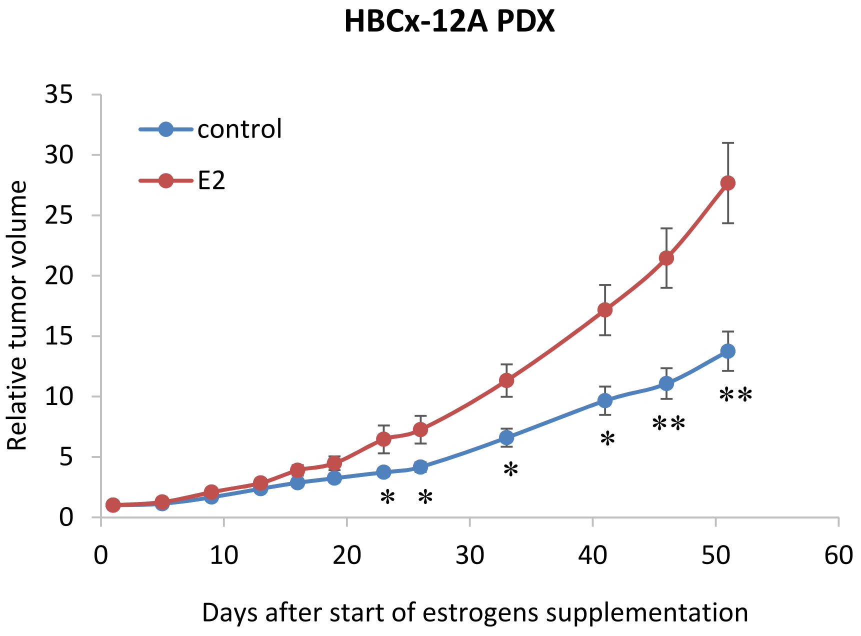

Figure S2

Supplementary Figure 2. Characterization of the in-house anti-ERα-36 antibody and of the HBCx-12A tumor

(a) GST-ERα-36 and GST-ERα-36ΔC were analyzed by SDS-PAGE followed by Western blot with our in-house anti-ERα-36 antibody (left panel), with the corresponding Coomassie-stained gel (right panel). * indicates the full length fusion proteins.
(b) HeLa cells transfected with the empty vector, pSG5-ERα, pCR1-ERα-46, pSG5-ERβ, pSG5Flag-ERα-36 or pSG5Flag-ERα-36ΔC were analyzed by Western blot using the anti-Flag, anti-ERα, anti-ERβ and anti-ERα-36 antibodies.
(c) Cama-1 cells were transfected with control siRNA duplexes or with 2 specific ERα-36 siRNA duplexes. On day 3, cells were re-transfected with the corresponding siRNAs. Cells were then lysed on the indicated dates and the lysates were tested for ERα-36 expression. Tubulin was used as a loading control.
(d) ERα-36 expression was evaluated in a wide range of human breast cell lines by Western blot using the anti-ERα-36 antibody. ERα and tubulin expression were also assessed using the corresponding antibodies.
(e) ERα-36 expression was evaluated in human patient derived xenografts (PDXs) as indicated in Figure S2D.
(f) ERα-36 expression was analyzed by immunohistochemical (IHC) staining on formalin-fixed human tumors from 2 PDXs: namely the HBCx-12A, expressing high level of ERα-36 and the HBCx-8, which do not express ERα-36. (x40 magnification).
(g) HBCc-12A cell lysate was assessed for ERα-36, ERα, PR and HER2 expression.
(h) HBCc-12A cells were treated for the indicated times with E_2 (10^{-8} M). The cell lysates were analyzed by Western blot for ERK and Akt activation by measuring their phosphorylated state using specific antibodies. The abundance in ERK and Akt proteins were measured using specific antibodies.
(i) HBCc-12A cells growth rate was monitored by the Incucyte real-time imaging system, under control conditions (vehicle) or after treatment with E_2 (10^{-8}M). The P-value was determined by the Student's test. *P<0.05.
(j) Tumor growth of HBCx-12A PDX was analyzed with or without estrogen for 51 days. For each group the relative tumor volume was measured. Each treatment group included 10 mice. Statistical significances of TGI were calculated using a Welch's t-test . **P<0.01

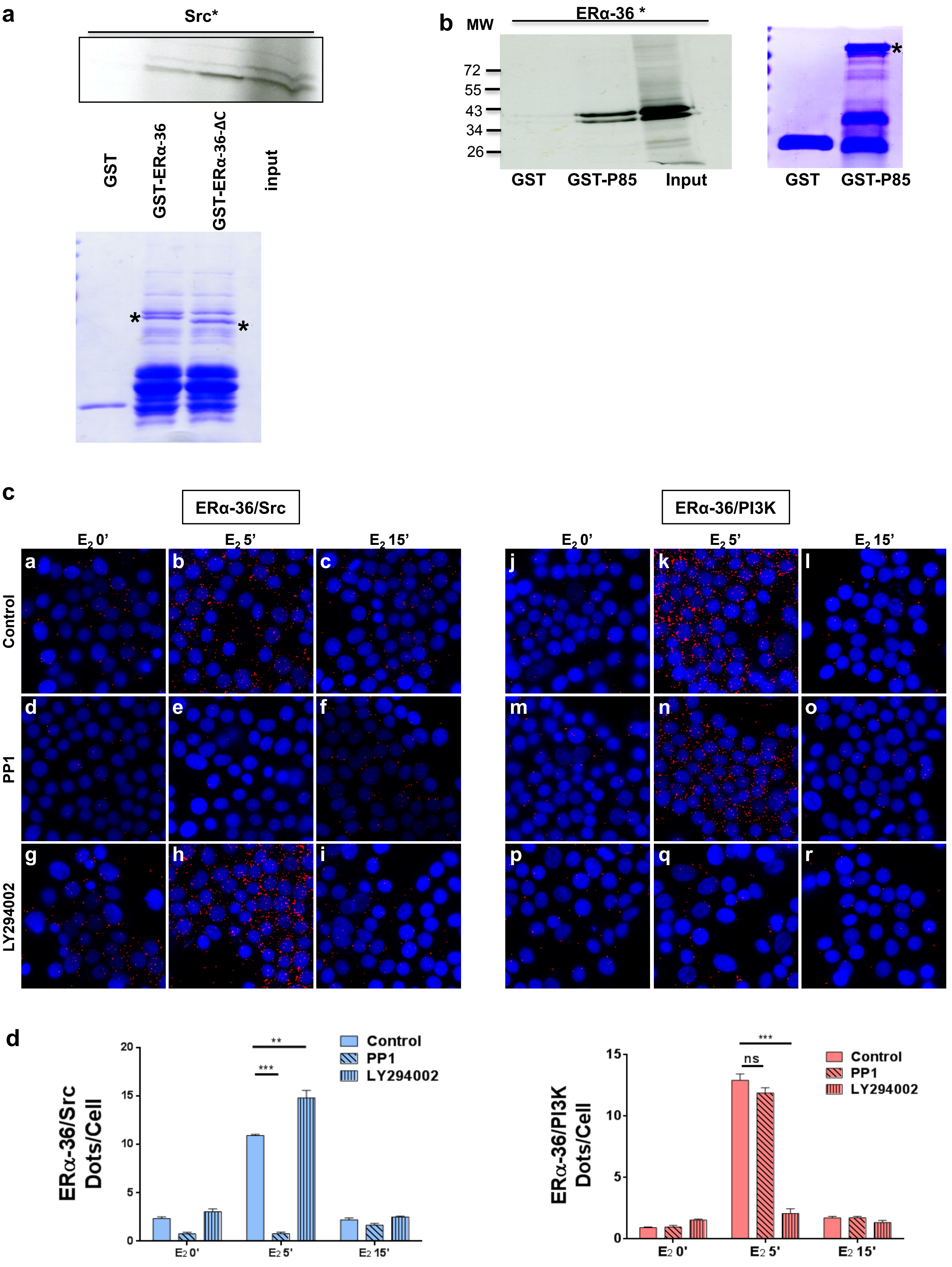
a
Src*
GST
GST-ERα-36
GST-ERα-36-ΔC
input
*
*
b
MW
ERα-36 *
72
55
43
34
26
GST
GST-P85
Input
GST
GST-P85
*
c
ERα-36/Src
ERα-36/PI3K
E2 0'
E2 5'
E2 15'
E2 0'
E2 5'
E2 15'
Control
PP1
LY294002
a b c j k l
d e f m n o
g h i p q r
d
ERα-36/Src Dots/Cell
0
5
10
15
20
**
***
Control
PP1
LY294002
E2 0'
E2 5'
E2 15'
ERα-36/PI3K Dots/Cell
0
5
10
15
***
ns
Control
PP1
LY294002
E2 0'
E2 5'
E2 15'

Figure S3

Supplementary Figure 3. Study of ERα-36/Src and ERα-36/PI3K interaction.

(a) GST pull down Assay was performed by incubating *in vitro*-translated ^{35}S-labeled Src (*) with GST, GST-ERα-36 and GST-ERα-36ΔC. The corresponding Coomassie-stained gel is shown in the lower panel. * indicates the full length fusion proteins.

(b) GST and GST-P85 of PI3K fusion proteins were incubated with *in vitro*-translated ^{35}S-labeled ERα-36 (*). The corresponding Coomassie-stained gel is shown in right panel. * indicates the full length fusion protein.

(c) HBCc-12A cells were treated in the presence or in the absence of the Src inhibitor PP1 (5µM) or the PI3K inhibitor LY294002 (20µM) for prior to the addition of E_2. After fixation, *in situ* PLA was performed with ERα-36-, Src-, and PI3K-specific antibodies. The detected dimers are represented by red dots. The nuclei were counterstained with DAPI (blue) (x63 magnification).

(d) Quantification of the number of signals per cell in Figure S3C was performed using computer-assisted analyses as reported in the Supplemental Experimental Procedures. The experiment was performed in triplicate, and this graph is representative of one of the experiments. The P-value was determined using the Student's test. ** P<0.01; *** P<0.001. NS: non significant.

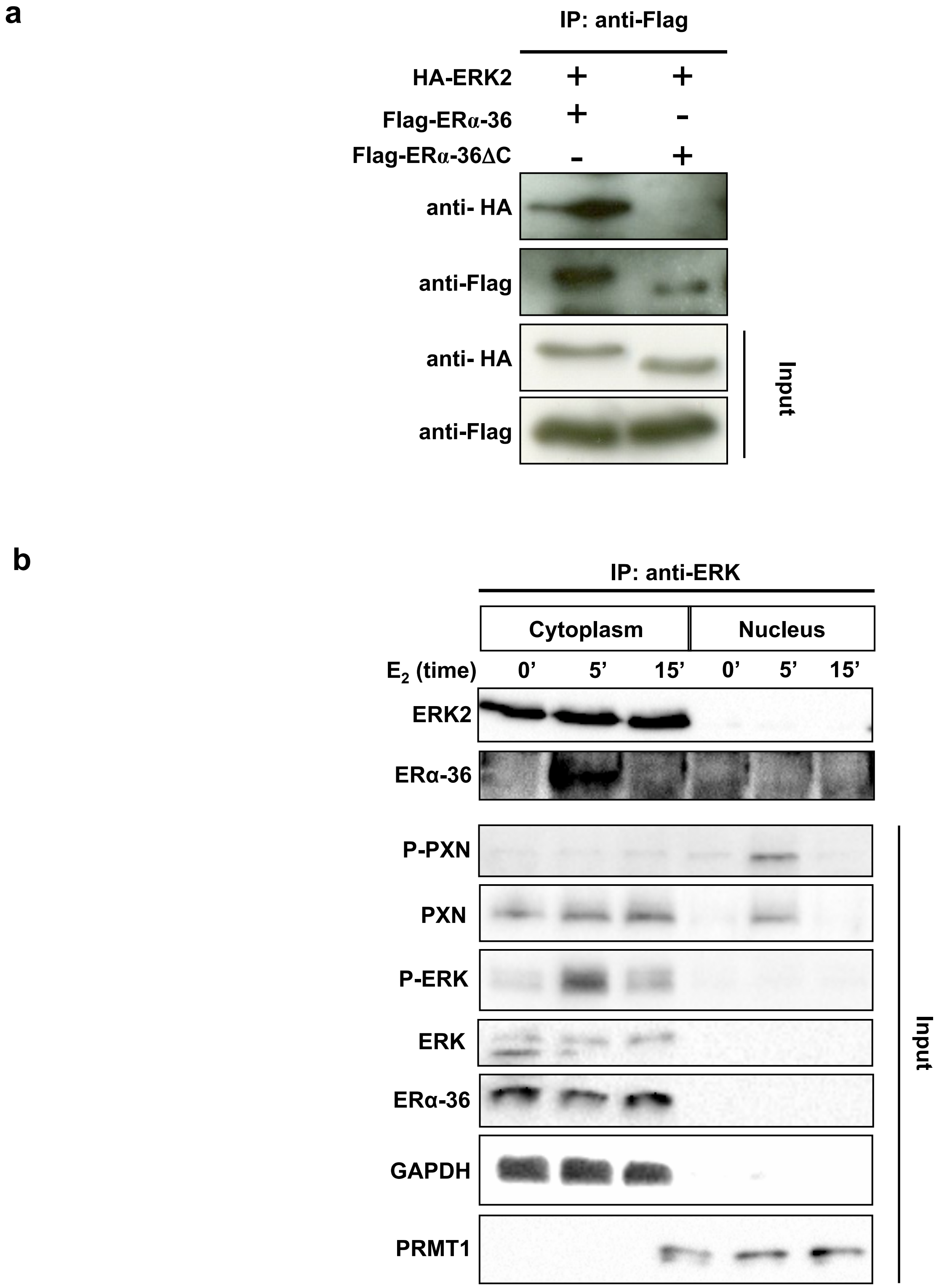
a
IP: anti-Flag
HA-ERK2 + +
Flag-ERα-36 + -
Flag-ERα-36ΔC - +
anti- HA
anti-Flag
anti- HA
anti-Flag
Input
b
IP: anti-ERK
Cytoplasm
Nucleus
E2 (time) 0' 5' 15' 0' 5' 15'
ERK2
ERα-36
P-PXN
PXN
P-ERK
ERK
ERα-36
GAPDH
PRMT1
Input

Figure S4

Supplementary Figure 4. Study of ERα-36/ERK2 interaction *in cellulo*.

(a) pSG5Flag-ERα-36, pSG5Flag-ERα-36ΔC and pCDNA3HA-ERK2 were overexpressed in HeLa cells. Cell lysates were immunoprecipitated with the anti-Flag antibody and the presence of ERα-36 and ERK2 were revealed by Western blot using the anti-Flag and anti-HA antibodies, respectively. The lower panel shows the expression of the different proteins in the input.

(b) Estrogen-deprived HBCc-12A cells were treated with E_2 (10^{-8}M) for the indicated times. Cytoplasmic and nuclear fractions were prepared at each time, then immunoprecipitated with an anti-ERK antibody followed by western blotting with anti-ERK and anti-ERα-36 antibodies. The quality of the fractionation was evaluated by western blot with the anti-GAPDH and PRMT1 antibodies. We also assessed the expression of ERK, P-ERK, PXN and P-PXN.

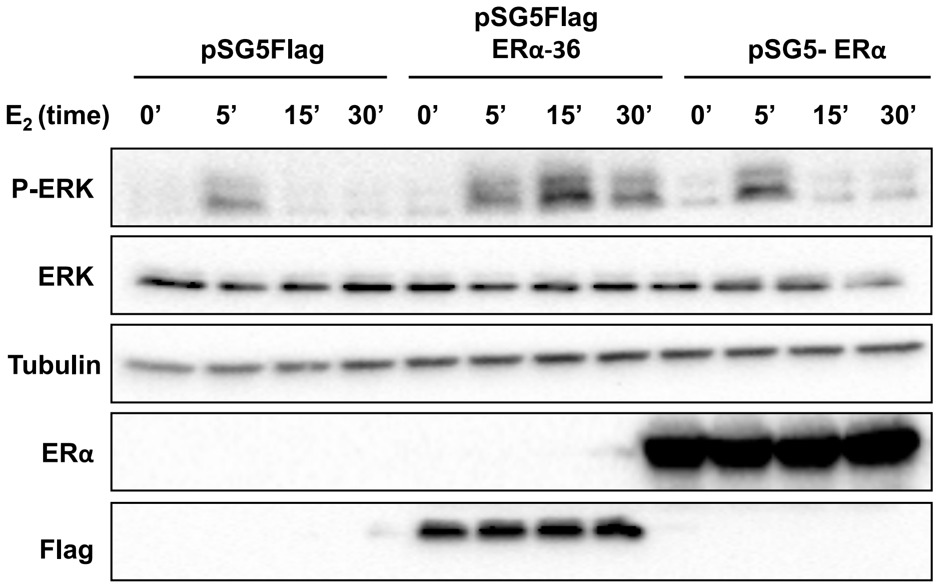
pSG5Flag
pSG5Flag ERα-36
pSG5- ERα
E2 (time)
0’ 5’ 15’ 30’ 0’ 5’ 15’ 30’ 0’ 5’ 15’ 30’
P-ERK
ERK
Tubulin
ERα
Flag

Figure S5

Supplementary Figure 5. Effect of ERα and ERα-36 expression on ERK phosphorylation

The pSG5-Flag or pSG5-Flag-ERα-36 vectors were transfected into HBCc-12A cells for 36 hr prior to E_2 activation. The cell extracts were analyzed for the expression of P-ERK, ERK, ERα, ERα-36 and tubulin expression.

.

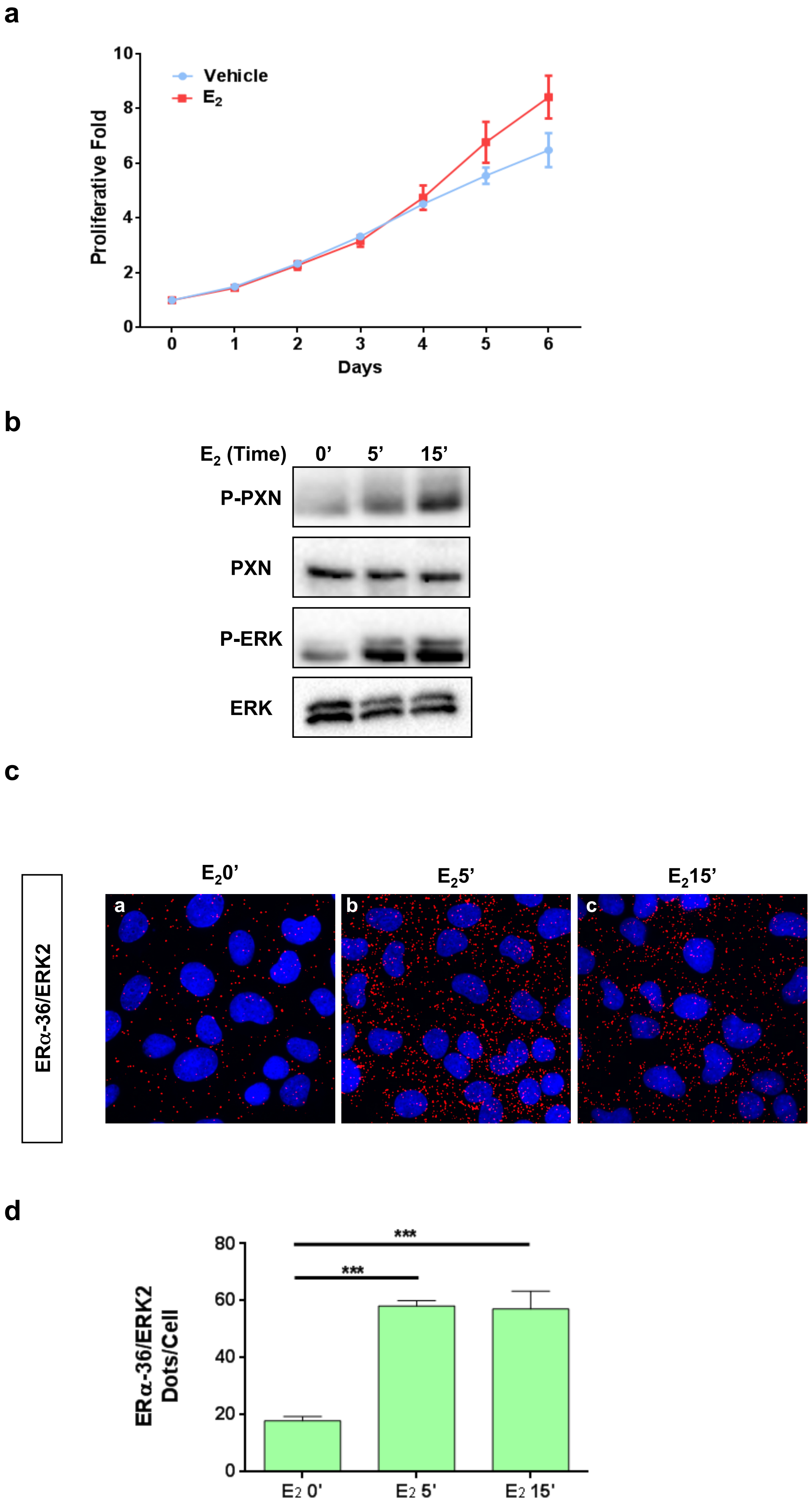
a
Vehicle
E2
Proliferative Fold
Days
0
1
2
3
4
5
6
2
4
6
8
10
b
E2 (Time)
0'
5'
15'
P-PXN
PXN
P-ERK
ERK
c
ERα-36/ERK2
E2 0'
E2 5'
E2 15'
a
b
c
d
ERα-36/ERK2 Dots/Cell
0
20
40
60
80
***
***
E2 0'
E2 5'
E2 15'

Figure S6

Supplementary Figure 6. ERα-36 signaling in HBL100 cells

(a) The growth rate of HBL100 cells was monitored using the Incucyte real-time imaging system in the absence or in the presence of E_2 (10^{-8}M). The experiment was performed in triplicate, and this graph is representative of one of the experiments.
(b) Steroid-depleted HBL100 cells were treated with E_2 (10^{-8}M) for the indicated times. Cell lysates were analyzed by Western blot for expression of P-ERK and P-PXN. ERK and PXN expression was also assessed.
(c) From the same experiment as depicted in Figure S5B, *in situ* PLA was performed to detect ERα-36/ERK2 interactions. The detected dimers are represented by red dots. The nuclei were counterstained with DAPI (blue) (x63 magnification).
(d) The quantification of the interactions detected in the PLA in Figure S5C was performed by counting the number of signals per cell using computer-assisted analyses, as reported in the Supplemental Experimental Procedures. The experiment was performed in triplicate, and this graph is representative of one of the experiments. The P-value was determined using the Student's test. *** P<0.001.

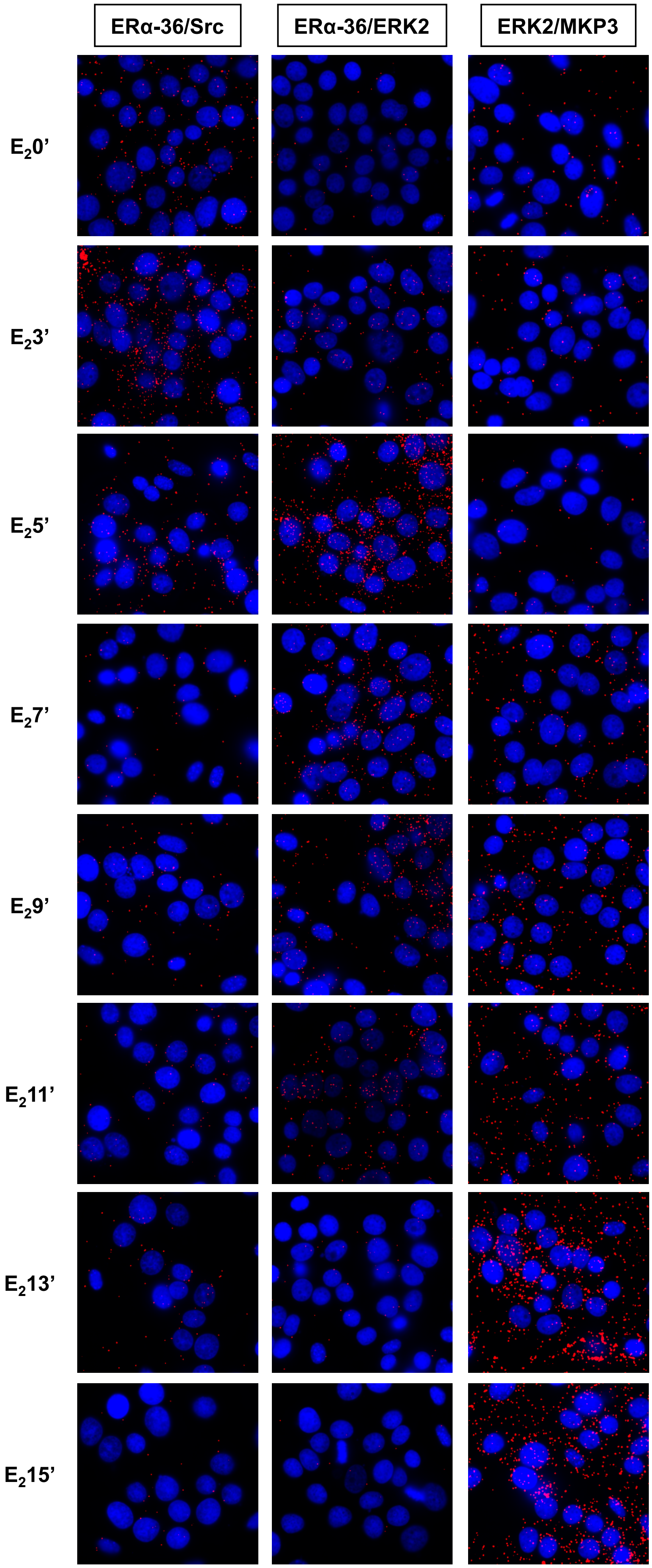
ERα-36/Src
ERα-36/ERK2
ERK2/MKP3
E2 0’
E2 3’
E2 5’
E2 7’
E2 9’
E2 11’
E2 13’
E2 15’

Figure S7

Supplementary Figure 7. Precise time course of the E_2/ERα-36-mediated signaling pathway.
HBCc-12A cells were treated with E_2 (10^{-8}M) for the indicated times. Then, ERα-36/Src, ERα-36/ERK2 and ERK2/MKP3 interactions were studied by PLA using the different couples of antibodies, as previously described. The detected dimers are represented by red dots. The nuclei were counterstained with DAPI (blue) (x63 magnification).

Supplementary Table 1: ***Residues in the D motif peptides which contribute for 4 kcal/mol and more to the binding free energy.*** For each structure listed in Table S3, we identified the residues of the bound peptide that contribute (in absolute value) for 4 kcal/mol or more to the binding free energy. For each complex, the number of the amino-acids, as given in Table S2, and the energetic contribution (in kcal/mol) are indicated. The known peptide binders differ in sequence, and do generally not conform exactly to the consensus $(R/K)_{2\text{-}3}X_{2\text{-}6}\text{-}\Phi_A\text{-}X\text{-}\Phi_B$ D motif. However, basic and hydrophobic residues consistently emerge in all structures as making important stabilizing interactions, with the important hydrophobic interactions situated in either N-terminal or C-terminal (or sometimes on both side) of the important basic arginine (R). From this energetic analysis, and consideration of the structure of peptide bound ERK2, we identified L297 in ERα-36 (in the sequence: $K_{293}KRIL_{297}NL_{299}$) as a likely candidate for making essential interactions..

**2GPH**		**4FMQ**	
L_{17}	-5.9	L_{436}	-4
R_{21}	-6.3	L_{439}	-4.9
L_{27}	-5.4	L_{444}	-5.8
L_{29}	-6.2	R_{448}	-7.3
**3TEI**		**2Y9Q**	
L_{714}	-6.7	M_{434}	-4.7
I_{717}	-5.2	L_{436}	-6.8
L_{722}	-5.4	P_{439}	-4.9
R_{726}	-4.4	L_{444}	-5.6
		R_{448}	-6.9
**3O71**		**4H3P**	
R_{1148}	-5.5	L_{714}	-7.3
P_{1149}	-5	I_{717}	-5.3
L_{1152}	-6.4	L_{722}	-5.8
I_{1154}	-4.2	R_{725}	-4
**4H3Q**		**2FYS**	
R_{5}	-4.4	R_{65}	-4.9
L_{9}	-4	L_{71}	-7.1
L_{12}	-5.8	R_{74}	-5.6
I_{14}	-4.9		

Supplementary Experimental Procedures

Cell Culture

We used a panel of human tumoral mammary cells, a gift from Jessie Auclair (CLB, Lyon). (Cama-1, T47D, ZR75.1, MDAMB361, BT20, Cal51, HCC38, HBL100, SKBR3, MDAMB157, MDAMB231, MDAMB436, MDAMB453 cells). All cell lines were regularly tested for mycoplasma contamination.

We also used frozen tumors from human breast patient's derived xenograft (PDX) from Dr Marangoni of Curie Institute, Paris. These PDX have already been characterized: HBCx-14, HBCx-10, HBCx-15, HBCx-17, HBCx-3, HBCx-8, HBCx-13A, HBCx-5, HBCx-12A, HBCx-29, HBCx-1, HBCx-1 (Marangoni et al, 2007).

The HBCx-12A was established by engrafting a triple negative tumor, and its molecular characterization has been previously published [1]. The HBCc-12A cell line was established from the HBCx-12A PDX. The HBCc-12A cell line and the HBCx-12A PDX were genotyped with the GenePrint® 10 System (#B9510, Promega) that allows co-amplification and three-color detection of nine human loci.

HBCx-12A cell line was grown at 37°C in DMEM Glutamax medium supplemented with 10% Fetal Bovine Serum, 1% Penicillin Streptomycine, 1% Hepes Buffer, 1% Sodium Pyruvate and 10 µg/ml Insulin (Novorapid).

Prior to performing treatment with estrogen ligands, cells were grown for 48 hr in phenol red-free medium supplemented with 10% charcoal-stripped serum (Biowest), in order to remove steroid hormones (steroid-depletion). The cells were then treated for different times with E_2 (Sigma) 10^{-8} M. When stated, cells were treated with the Src inhibitor PP1, the PI3K inhibitor LY294002, or with the MEK inhibitor U1026 (Calbiochem).

Plasmids

ERα-36 cDNA was cloned into the pSG5Flag and the pGEX-4T1 plasmids. The ERα36 CTD was cloned into a pSG5-Flag vector (pSG5Flag-ERα-36-CTD). The pSG5Flag-ERα-36 was used for mutagenesis to generate the following mutants: V288A/L295A, E180A, E180A/R221A, and L297A. The pCDNA3 ERK2-HA and pCDNA3-Src plasmids were purchased from Addgene. ERK2 cDNA was subsequently cloned into a pGEX-4T1 vector for recombinant protein production. pGEX-4T1-P85 (PI3K) plasmids are gifts from Dr G. Castoria.

Nes Cloning:

PCR was carried out on a pSG5-PRMT1 plasmid. Primers were designed so that the NES of ERα-36 was in phase with the PRMT1 ORF at its 5' end. The PCR product was purified with QIA Quick Gel Extraction Kit and was subsequently cloned into a pGEMT-Easy vector (Promega). The construct was sequenced (Beckman Coulter Genomics) and verified for the presence of the NES in phase with the PRMT1 ORF. Sub cloning was then performed in a pSG5Flag vector using BamHI and Xho1 Restriction Sites.

Primer Sequences

BamH1- ERα-36 NES – PRMT1 Forward Primer
5'GGATCCgtagaagcaaagaagagaatcctgaacttgATGGCGGCAGCCGAGGCCGCGAACTGCATCATG 3'

XhoI – PRMT1 Reverse Primer
5' CTCGAGtcagcgcatccggtagtcggtggaacaagagagctcacacagctgac 3'

Antibodies

Antibody	Company	Species	Dilution for WB	Dilution for PLA	Dilution for IF	Dilution for IHC	Dilution for IP
Flag *(E1B11)*	Euromedex	Mouse	1:1000		1 :250		
Tubulin *(T6074)*	Sigma	Mouse	1:10000				
Src, B12 *(sc-8056)*	Santa Cruz	Mouse		1:200			
ERα-36	In-house (Covalab, Lyon)	Rabbit	1:1000	1:100		1:50	
PI3K *(ab86714)*	Abcam	Mouse		1:300			
p42/44 MAPK *(4376)*	CST	Rabbit	1:1000				
p-P42/44Thr 202/Tyr204 MAPK *(4370)*	CST	Rabbit	1:1000		1 :100	1 :400	
ERK2 D2 *(1647)*	Santa Cruz	Mouse	1:1000	1:100			
ERK2	Abcam	Rabbit					1:100
MKP3 *(ab76310)*	Abcam	Rabbit	1:1000	1:100			
PXN *(5574)*	Santa Cruz	Rabbit	1:1000				
P-PXN Ser^{126} *(44-1022G)*	Invitrogen	Rabbit	1:1000		1 :100	1 :300	
Cyclin D1 *(ab16663)*	Abcam	Rabbit	1:200				
AKT *(9272S)*	CST	Rabbit	1 :1000				
P-AKT Ser^{473} *(9271L)*	CST	Rabbit	1 :1000				
HA *(H6908)*	Sigma	Rabbit	1:1000				
HER2 *(ab16901)*	Abcam	Mouse	1:500				
PR *(C1A2)*	CST	Rabbit	1:1000				
PRMT1	Millipore	Rabbit	1:2000				
ERα *(60C)*	Millipore	Rabbit	1 :1000				

Supplementary Table 2: List of the antibodies used in the current work

Modelling data: Analysis of ERK2/peptide structures and identification of important amino acids.
We analyzed 8 crystal structures of ERK2 bound to peptide that contain D motifs (see list in Table S1). Hydrogen atoms were added using the HBUILD facility [2] in the CHARMM program [3]. The structures were energy minimized and free energy decomposition [4] of the minimized structure was used to estimate the contribution of each amino acid to the binding free energy. Free energy decomposition allows a semi-quantitative estimate of the contribution of amino acids to the stability of a complex, and is well suited to identify amino acids essential for complex formation [4]. Amino acids detailed in Table S1 are in bold red.

PDBid	Peptide sequence	Reference
2GPH	R$_{16}$**L**QER**R**GSNVA**L**M**L**DC$_{31}$	5
4FMQ	**L$_{436}$**SS**L**AASS**L**AKR**R**QQ$_{450}$	6
3TEI	P$_{712}$Q**L**KP**I**ESSI**L**AQR**R**VR$_{728}$	6
2Y9Q	**M$_{434}$**K**L**SP**P**SKSR**L**ARR**R**AL$_{450}$	6
3O71	**R$_{1148}$P**PD**L**W**I**H$_{1155}$	7
2FYS	R$_{64}$**R**LQKGN**L**PV**R$_{74}$**	8
4H3P	P$_{712}$Q**L**KP**I**EASI**L**AA**R**RV$_{727}$	9

Supplementary Table 3: Structures used for the interaction between ERK2 and D motif peptides. Amino acids detailed in supplementary Table 1 are in bold red.

Patient-derived xenografts (PDXs)

The HBCx-12A PDX was established from a primary triple-negative breast cancer (ERα-, PR-, HER2-) with the patient's informed consent, as described previously [1]. The PDXs were engrafted in 10-week old female Swiss nude mice, purchased from Charles River (L'Arbresle, France) and maintained under specific pathogen-free conditions. Their care and housing were done in accordance with institutional guidelines approved by the French Ethical Committee [1]. The ER+, HER2+ and triple-negative status were confirmed in the PDX models by immunohistochemistry (IHC) [1, 10]. When tumors reached a volume of 60 to 200 mm3, mice were individually identified and randomly assigned to the control or treated groups (10 mice per group), and the treatments were started. Estradiol (E_2, Sigma-Aldrich, Saint-Quentin-Fallavier, France) was diluted in their drinking water (8 µg/ml). A Welch's t-test was used for the statistical analysis of tumor growth in treated E_2 versus control mice.

Glutathione transferase (GST) pull-down assay

ERα-36 and ERα-36ΔC expression plasmids were transcribed and translated *in vitro* using T7-coupled reticulocyte lysate in the presence of [^{35}S] methionine. Labeled proteins were incubated with 10 µg of purified recombinant GST-fusion proteins in 200 µl of binding Buffer (Tris 20 mM pH7.4, NaCl 0.1 M, EDTA 1 mM, Glycerol 10%, Igepal 0,25%) with 1 mM DTT and 1% milk) for 2 hr at room temperature. After washing, bound proteins were resolved on SDS-polyacrylamide gel electrophoresis (PAGE), and visualized by autoradiography.

Transfections

The siRNA sequences targeting ERα-36 correspond either ERα-36 to the 3'UTR (GGUCAAAGAUCAAGAUCAAdTdT) (Nucleotides 1631 – 1643) or to the ERα-36 and ERα conserved sequence (GAAUGUGCCUGGCUAGAGAdTdT) (Nucleotides 852 – 870).

SiRNA directed against MKP3 (DUSP6) were purchased from Qiagen GeneSolution (Cat No. GS1848). 50 nM of specific siRNAs or the scrambled siRNA (Eurogentec) were transfected into HBCc-12A cells (1 x 10^6) using lipofectamine 2000 reagent (Invitrogen) according to the manufacturer's guidelines. The proteins were analyzed 72 hr after transfection.

Plasmids transfection was performed using the Xtreme gene agent following the manufacturer's instructions (Roche). Proteins were analyzed 48 hr after transfection.

HBCc-12A cells were infected with Histone H2B fused to GFP (gift from Dr Y. Mikaelian, CRCL, Lyon, France).

Subcellular fractionation

2 million cells were seeded in 10 cm plates. rinsed twice with ice cold PBS and were then incubated on ice to swell for 10 minutes with 4ml LS 'Low salt' Buffer (20 mM HEPES pH 7.8, 5 mM Potassium Acetate, 0.5 mM Magnesium Chloride, 0.5 mM DTT), supplemented with phosphatase inhibitors (1 mM NaF, 1 mM Na_3VO_4, 1 mM β-glycerophosphate) and protease inhibitor tablets (Roche Molecular Biochemicals). All the buffer was aspirated and cells were scraped and disrupted with 25 strokes in a Dounce homogenizer (1-ml Dounce tissue grinder; Wheaton, Millville, NJ) using a loose-fitting pestle Nuclei were pelleted at 1,500 g for 3 min and the supernatant was recentrifuged at 14,000 g for 20 min at 4°C. Nuclei were lysed with RIPA buffer (50 mM Tris HCl, pH 8, 150 mM NaCl, 1 mM EDTA, 1% NP-40, 0.25% deoxycholate) supplemented with protease inhibitor tablets (Roche Molecular Biochemicals) and phosphatase inhibitors (1 mM NaF, 1 mM Na_3VO_4, 1 mM β-glycerophosphate).

Proliferation assay

Cells were plated in quadruplicate in 96-well plates at a density of 2000 cells per well. After 6h, the ligands were added into the medium, and subsequently every two days. Growth curves were constructed by imaging plates using the Incucyte Zoom (Essen BioScience, Ann Arbor, USA). The growth curves were built from confluence measurements for HBL100 cells, and from counting fluorescent nuclei for HBCc-12A cells, acquired during round-the-clock kinetic imaging. Each experiment was performed in quadriplicate.

Immunoprecipitation and Western blot

To study the effect of ERα ligands on ERα-36 signaling, the cells were treated for different periods of time with E_2. After treatment, cells were lysed using RIPA buffer. Protein extracts were incubated with primary antibodies overnight at 4°C with in a shaking incubator. Protein A-Agarose beads were added and the solution was incubated 1 hr at 4°C. The immunoprecipitates were separated on SDS-PAGE. The proteins were visualized by enhanced chemiluminescence (ECL) (Roche Molecular Biochemicals).

Immunofluorescence

HBCc-12A cells (9 x 10^4) were grown on coverslips in 12-well plates. Cells were fixed in methanol for 2 min, washed twice in PBS and incubated with primary antibodies for 1 hr at 37°C. After PBS washes, the cells were incubated for 30 min at 37°C with the secondary antibody Alexa Fluor 468 from Molecular Probes (1:3000) in Dako diluent, then washed in PBS and mounted on glass slides in mounting solution (Dako), and visualized using a fluorescent microscope.

Immunohistochemical (IHC) staining

Paraffin embedded tumour tissues fixed in formalin were used for analysis. After deparaffinization and rehydratation, tissue sections were boiled in 10 mM citrate buffer pH 6 at 97°C for 40 min. The slides were then incubated in 5% hydrogen peroxide in sterile water to block the activity of endogenous peroxidases. The slides were then incubated at room temperature for 1 hr with the primary antibodies. The slides were subsequently incubated with a biotinylated secondary antibody bound to a streptavidin peroxidase conjugate (Envision Flex kit Ref: K800021-2, Dako). Bound antibodies were revealed by adding the substrate 3, 3-diamino benzidine. Sections were counterstained with haematoxylin.

Proximity ligation assay (PLA)

PLAs performed on fixed cells were revealed by adding fluorescent probes, while PLAs performed on fixed tissues were revealed by adding peroxidase-labeled probes.

Fluorescence detection

Cells were grown on coverslips in 12-well plates, and then fixed in methanol for 2 min, before being washed twice in PBS. The cells were initially saturated using the blocking solution, then different couples of primary antibodies (rabbit and mouse in our case) were added and incubated 1 hr at 37°C. After washes, the PLA minus and plus probes (containing the secondary antibodies conjugated with complementary oligonucleotides) were added and incubated 1 hr at 37°C. Following the ligation of the oligonucleotides, nucleotides and a polymerase were added to the solution to initiate, a rolling-circle amplification (RCA) reaction, using the ligated circle as a template for 100 min at 37°C. The amplification solution contained fluorescent-labeled oligonucleotides, which hybridized to the RCA product. Following hybridization, the samples were mounted with Duolink II Mounting Medium containing 4',6-diamidino-2-phenylindole (DAPI), and then visualized under fluorescent microscopy.

Bright-field detection

Fixed tumor tissues were initially incubated in a hydrogen peroxide solution, for 5 min at room temperature, to avoid peroxidase quenching. The following steps were to those described above. For the detection, the probes were labeled with horseradish peroxidase after two washes in high purity water. A nuclear staining solution was added onto the slides and incubated 2 min at room temperature. After washing the slides 10 min under running tap water, the samples were dehydrated in ethanol and in xylene. Samples were mounted in non-aqueous mounting medium and then analyzed using a bright-field microscope.

Image acquisition and analysis

The hybridized fluorescent slides were viewed under a Nikon Eclipse Ni microscope. Images were acquired under identical conditions at x63 magnification. Image acquisition was performed by imaging DAPI staining at a fixed Z Position while a Z stack of +/- 5µm at 1 µm intervals was carried out. The final image was stacked to a single plane before further quantification. On each sample, at least one hundred cells were counted. Analyses and quantifications of these samples were performed using Image J software (free access). PLA dots were quantified on 8-bit images using the 'Analyse Particles'command, while cell numbers were numerated using the cell counter plugin.
IHC images were acquired using a Leica DMRB microscope at x40 magnification and PLA dots were quantified as described above.

Supplementary References

1. Marangoni,E. *et al.* A new model of patient tumor-derived breast cancer xenografts for preclinical assays. *Clin. Cancer Res.* **13**, 3989-3998 (2007).

2. Brunger,A.T. & Karplus,M. Polar hydrogen positions in proteins: empirical energy placement and neutron diffraction comparison. *Proteins* **4**, 148-156 (1988).

3. Brooks,B.R. *et al.* CHARMM: the biomolecular simulation program. *J. Comput. Chem.* **30**, 1545-1614 (2009).

4. Lafont,V., Schaefer,M., Stote,R.H., Altschuh,D., & Dejaegere,A. Protein-protein recognition and interaction hot spots in an antigen-antibody complex: free energy decomposition identifies "efficient amino acids". *Proteins* **67**, 418-434 (2007).

5. Zhou,T., Sun,L., Humphreys,J., & Goldsmith,E.J. Docking interactions induce exposure of activation loop in the MAP kinase ERK2. *Structure.* **14**, 1011-1019 (2006).

6. Garai,A. *et al.* Specificity of linear motifs that bind to a common mitogen-activated protein kinase docking groove. *Sci. Signal.* **5**, ra74 (2012).

7. Ma,W. *et al.* Phosphorylation of DCC by ERK2 is facilitated by direct docking of the receptor P1 domain to the kinase. *Structure.* **18**, 1502-1511 (2010).

8. Liu,S., Sun,J.P., Zhou,B., & Zhang,Z.Y. Structural basis of docking interactions between ERK2 and MAP kinase phosphatase 3. *Proc. Natl. Acad. Sci. U. S. A* **103**, 5326-5331 (2006).

9. Gogl,G., Toro,I., & Remenyi,A. Protein-peptide complex crystallization: a case study on the ERK2 mitogen-activated protein kinase. *Acta Crystallogr. D. Biol. Crystallogr.* **69**, 486-489 (2013).

10. Reyal,F. *et al.* Molecular profiling of patient-derived breast cancer xenografts. *Breast Cancer Res.* **14**, R11 (2012).
